# Temporal trends in the planetary health diet index and its association with cardiovascular, kidney, and metabolic diseases: A comprehensive analysis from global and individual perspectives

**DOI:** 10.1016/j.jnha.2025.100520

**Published:** 2025-02-21

**Authors:** Haoxian Tang, Xuan Zhang, Nan Luo, Jingtao Huang, Qinglong Yang, Hanyuan Lin, Mengyue Lin, Shiwan Wu, Jiasheng Wen, Jianan Hong, Pan Chen, Liwen Jiang, Yequn Chen, Xuerui Tan

**Affiliations:** a Shantou University Medical College, Shantou, Guangdong, China; b Department of Cardiology, The First Affiliated Hospital of Shantou University Medical College, Shantou, Guangdong, China; c Department of Bone & Joint Surgery, Peking University Shenzhen Hospital, Shenzhen, Guangdong, China; d Department of Psychiatry, Shantou University Mental Health Center, Shantou, Guangdong, China; e Department of Sports Medicine and Rehabilitation, Peking University Shenzhen Hospital, Shenzhen, Guangdong, China; f Department of Urology, The Second Affiliated Hospital of Shantou University Medical College, Shantou, Guangdong, China; g Clinical Research Center, The First Affiliated Hospital of Shantou University Medical College, Shantou, Guangdong, China; h Human Phenome Institute, Shantou University Medical College, Shantou, Guangdong, China; i Guangdong Engineering Research Center of Human Phenomics, Shantou, Guangdong, China

**Keywords:** Planetary health diet index, Cardiovascular, kidney, and metabolic diseases, Global burden of disease, Global dietary database, National health and nutrition examination survey

## Abstract

**Background:**

Diet plays a critical role in human health and environmental sustainability, particularly in cardiovascular, kidney, and metabolic (CKM) diseases. However, the variations in the Planetary Health Diet Index (PHDI) across populations, regions, and over time, as well as its association with CKM disease burdens, remain insufficiently explored.

**Methods:**

We assessed PHDI scores using data from 185 countries (1990–2018) from the Global Dietary Database, examining demographic characteristics and temporal trends. The Global Burden of Disease Study was used to evaluate the associations between PHDI and CKM disease burdens, including incidence, prevalence, mortality, and disability-adjusted life years. CKM syndrome was defined by the American Heart Association. Individual-level data from the National Health and Nutrition Examination Survey (NHANES) were also used to assess the impact of PHDI on CKM risks and mortality.

**Results:**

From 1990 to 2018, while overall PHDI scores remained relatively stable between sexes, the composition of PHDI scores shifted across different age groups. In 2018, the mean PHDI score was 42.80 (95% uncertainty interval [UI] 42.49–46.50) for males and 44.65 (95% UI 44.53–47.82) for females. Higher PHDI scores were observed among females, older adults, urban residents, individuals with higher education, and those from South Asia. Globally, consumption of red/processed meat, saturated oils/trans fats, and added sugars substantially exceeded the EAT-Lancet Commission’s reference values. Higher PHDI scores were generally associated with lower CKM disease burdens, although these associations varied by disease subtype. In individual-level analysis, including 45,460 NHANES participants (weighted mean age: 47.21 years, 51.4% female), each 10-point increase in PHDI was linked to a 13.7% reduction in stage 3/4 CKM syndrome risk, an 11.1% reduction in stage 4 CKM syndrome risk, and lower incidences and mortality rates for cardiovascular diseases, metabolic diseases, and chronic kidney disease.

**Conclusions:**

From 1990 to 2018, significant changes occurred in the components of the PHDI, with notable variations by demographics and region. Higher PHDI scores may reduce CKM disease burdens, warranting further investigation into specific disease subtypes.

## 1 Introduction

The world faces urgent challenges related to food systems, which contribute approximately 26% of anthropogenic greenhouse gas emissions and excessive land and freshwater use [1]. Currently, food insufficiency remains a significant issue, and the burden of related diseases due to poor dietary quality is continuously rising. Diet is intricately linked to human health and environmental sustainability [[1], [2], [3]]. The EAT-Lancet Commission proposed a significant framework that integrates the scientific objectives of healthy diets and sustainable food systems, aiming to identify a dual-benefit diet that promotes health and environmental sustainability while supporting a growing population and mitigating environmental impacts. The Planetary Health Diet Index (PHDI) was established based on this framework [2,4]. The PHDI assesses dietary quality by evaluating the intake of 14 food groups, in line with the recommended ranges established by the EAT-Lancet Commission. Its scoring criteria are derived from dose-response relationships between these food groups and major chronic diseases, based on meta-analyses and large cohort studies, ensuring an accurate reflection of dietary health impacts.

Dietary risk plays a critical role in the development of cardiovascular, kidney, and metabolic (CKM) diseases. From 1990 to 2021, poor diet (For example, a diet low in fruits, high in red/processed meats, with specific definitions available at https://www.healthdata.org/gbd/methods-appendices-2021/dietary-risks) consistently ranked as the second leading risk factor for cardiovascular disease (CVD), diabetes mellitus (DM), and the third or fourth for chronic kidney disease (CKD). In 2021, 31.3% of CVD, 24.2% of DM, and 17.9% of CKD burdens were attributed to poor diet [5,6]. CKM diseases share common underlying mechanisms and interact at the pathophysiological level, creating a harmful cycle that perpetuates disease progression. This interaction leads to an increasing incidence of multi-organ dysfunction and adverse cardiovascular outcomes [[7], [8], [9]]. Recognizing this complex relationship, the American Heart Association (AHA) recently introduced the concept of CKM syndrome-a comprehensive disease state aimed at enhancing communication among scientists and stakeholders, identifying individuals at high risk for morbidity and mortality, and initiating preventive strategies before end-organ damage occurs [8].

Previous studies on the PHDI were limited to a few countries, leaving global variations by demographics (age, sex, education, urbanicity), regions, and temporal trends largely unexplored [4,[10], [11], [12]]. No research has examined the CKM disease burden from both global and individual perspectives [[13], [14], [15], [16]]. The current study addressed these gaps by using data from the Global Dietary Database (GDD) to describe demographic patterns and temporal trends in PHDI across 185 countries. We also assessed the association between PHDI and the global burden of CKM diseases using data from the Global Burden of Disease (GBD) study. Additionally, individual-level data from the National Health and Nutrition Examination Survey (NHANES) were used to evaluate the association between PHDI and the incidence and mortality risk of CKM diseases.

## 2 Methods

### 2.1 Data source and data collection

**Fig. 1:**
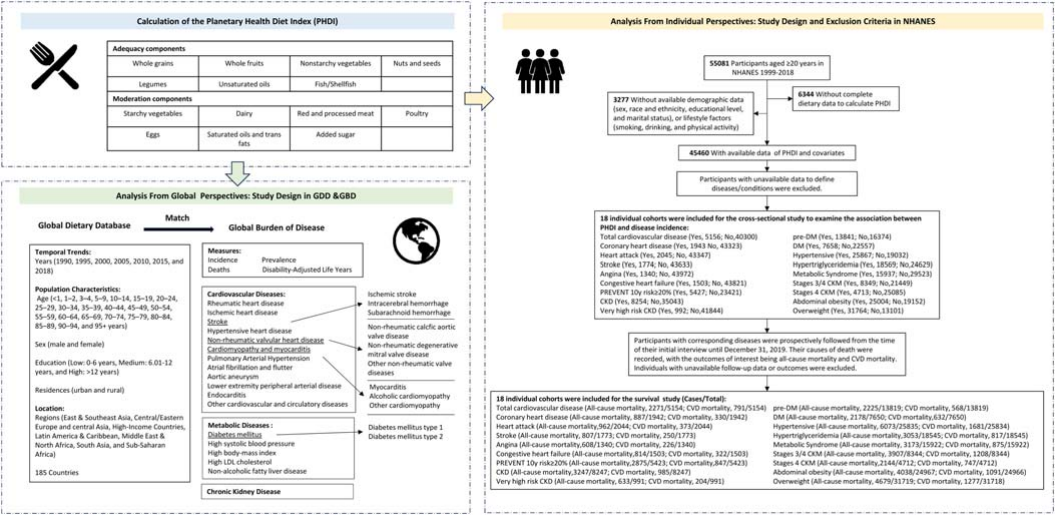
Study process and design. Abbreviations: CKD, Chronic Kidney Disease; CKM, Cardiovascular, Kidney, and Metabolic Diseases; GDD, Global Dietary Database; GBD, Global Burden of Disease; NHANES, National Health and Nutrition Examination Survey.

The specific study design was illustrated in Fig. 1.

The global food intake data for the current study were obtained from the GDD. Dietary survey data were collected from 185 countries in 1990, 1995, 2000, 2005, 2010, 2015, and 2018 through systematic online searches and direct communication with researchers and government authorities. Priority was given to surveys that were nationally or subnationally representative and employed standardized methods, such as 24 -h dietary recalls or food frequency questionnaires [[17], [18], [19]]. Data were extracted using consistent protocols and stratified by age (<1, 1–2, 3–4, 5–9, 10–14, 15–19, 20–24, 25–29, 30–34, 35–39, 40–44, 45–49, 50–54, 55–59, 60–64, 65–69, 70–74, 75–79, 80–84, 85–89, 90–94, and 95+ years), sex (male and female), education (low: 0–6 years, medium: 6.01–12 years, high: >12 years), and residence (urban, rural). A Bayesian model was applied to estimate mean intakes and associated uncertainties, with adjustments for energy intake to ensure comparability across populations and minimize measurement errors. During the data collection process for the GDD, dietary intakes were adjusted based on age-specific energy levels as follows: 700 kcal/day for individuals aged 0 to <1 year, 1000 kcal/day for ages 1 to <2 years, 1300 kcal/day for ages 2–5 years, 1700 kcal/day for ages 6–10 years, 2000 kcal/day for ages 11–74 years, and 1700 kcal/day for ages 75 years and older [20].

We further utilized data from the GBD 2021 to match the incidence, prevalence, mortality, and disability-adjusted life years (DALYs) of CKM diseases at a global level, by year, age (including only age≥15), and sex. GBD 2021, coordinated by the Institute for Health Metrics and Evaluation (IHME), covered different countries and territories from 1990 to 2021. It comprehensively assessed 371 diseases and injuries, 288 causes of death, and 88 risk factors contributing to disease burden. The methodological framework and principles of GBD 2021 have been extensively detailed in previous publications [5,21]. Additionally, we calculated the sociodemographic index (SDI), which evaluates social and economic conditions impacting health by computing the geometric mean of lag-distributed income, average years of schooling, and fertility rate [21].

In individual-level analysis, we used nationally representative data from the United States. The NHANES was conducted by the Centers for Disease Control and Prevention (CDC) and the National Center for Health Statistics (NCHS) and assessed the health and nutritional status of the U.S. civilian population [22,23]. NHANES employed a multistage, continuous cross-sectional survey design (conducted biennially), collecting data through home interviews and mobile examination centers. Dietary intake was assessed using two 24-hour recalls linked to the U.S. Department of Agriculture’s Food and Nutrient Database. The study was approved by the Institutional Review Board of the NCHS, and informed consent was obtained from all participants. In brief, the current study included 45,460 adults aged ≥20 years with available data on PHDI and relevant covariates (Fig. 1).

### 2.2 Calculation of the PHDI

The calculation method for the PHDI was detailed in Supplementary Table S1. The PHDI included assessments of 14 food groups: whole grains, whole fruits, nonstarchy vegetables, nuts and seeds, legumes (including nonsoy legumes and soybean/soy foods), unsaturated oils, fish/shellfish, starchy vegetables, dairy, red/processed meat, poultry, eggs, saturated oils/trans fats, and added sugars [24,25]. Each group was assigned a score from 0 to 10 using a proportional scoring method, with higher scores reflecting better adherence to recommended intake levels. Dietary consumption levels were standardized to 2500 kcal/day to derive the PHDI scores, in accordance with recommendations from the EAT-Lancet Commission and consistent with previously published research [25,26]. The total PHDI score, ranging from 0 to 140, was calculated by summing the scores across all food groups.

Notably, the original PHDI mainly consisted of “Moderation” components, which receive a score of 10 when intake is low. However, a score of 10 is also assigned when intake equals zero, potentially misclassifying malnourished individuals in resource-limited settings as having a healthy and sustainable diet [27,28]. To address this, we applied bidirectional scoring to “Moderation” components (excluding saturated oils/trans fats and added sugars) as outlined by Zhang et al. [24]. For example, for foods like starchy vegetables, dairy, red/processed meat, poultry, and eggs, we no longer use the median intake as the minimum threshold (where intake below the median was assigned a score of 10). Instead, we set zero as the minimum value, with proportional scores gradually decreasing between recommended intake levels and zero intake/maximum reference value. Additionally, in the GDD, nonsoy legumes and soybean/soy foods were combined for scoring [20]. Poultry intake was not reported, resulting in a theoretical PHDI score range in the GDD of 0 to 130.

### 2.3 Collection and definition of study outcomes

In the global-level analysis, we extracted data on incidence, prevalence, mortality, and DALYs for 12 subcategories and 9 detailed classifications of CVD, as well as 5 subcategories and 2 detailed classifications of metabolic diseases and CKD (Figure 1) [29]. Further details on disease definitions and data collection methodologies can be found on the GBD official website: https://www.healthdata.org/gbd/methods-appendices-2021.

In the individual-level analysis using NHANES data, CKM syndrome and its components, along with all-cause and CVD mortality among participants with corresponding diseases were considered as study outcomes (Fig. 1). CKM syndrome was defined by the co-occurrence of subclinical or clinical CVD, CKD, and metabolic disorders, stratified into four stages. The specific criteria for each stage are detailed in Supplementary Table S2 [30]. In brief, clinical CVD was defined as a history of coronary heart failure, coronary artery disease, heart attack, or stroke, while subclinical CVD was characterized by a ≥20% predicted 10-year CVD risk or the presence of very high-risk CKD. The 10-year CVD risk was estimated using the newly developed AHA PREVENT Equations [[30], [31], [32]], which incorporate CKM health parameters (Supplementary Table S3). According to the Kidney Disease: Improving Global Outcomes (KDIGO) Clinical Practice Guideline for the Management of Glomerular Diseases, CKD was defined and staged based on the estimated glomerular filtration rate and the albumin-to-creatinine ratio, as described in our previous work [33]. Metabolic disorders were classified as overweight, abdominal obesity, DM, pre-DM, hypertension, hypertriglyceridemia, and metabolic syndrome (Supplementary Table S2).

Participants with corresponding diseases were prospectively followed from their initial interview until December 31, 2019, with causes of death recorded for all-cause and CVD mortality. Using the International Classification of Diseases, 10th Revision (ICD-10), we analyzed deaths from all causes and defined CVD mortality as deaths due to heart diseases (I00–I09, I11, I13, I20–I51) [33].

### 2.4 Assessment of covariates

In the analysis of the association between the PHDI and disease burden at a global level, adjustments were made solely for year, age, and sex due to limitations in data availability that prevented further adjustment for other covariates. For the individual-level analysis, the following covariates were included: NHANES cycles, age, sex, race and ethnicity, educational level, marital status, smoking status, drinking status, and physical activity (PA). Age was incorporated into the model as a continuous variable, while sex was categorized as female and male. Race and ethnicity were classified as non-Hispanic White or others. Educational level was grouped into three categories: less than high school, high school or equivalent, and above high school. Marital status was defined as being married or others. Smoking and drinking status were categorized based on the presence or absence of these behaviors. PA was quantified as the total time spent weekly on occupational, household, recreational, and transportation activities, and was expressed in metabolic equivalent (MET) hours per week [22,34].

### 2.5 Statistical analysis

In the global-level analysis, PHDI score from 1990 to 2018 were initially described and categorized by age, sex, education, residence, and region. Subsequently, the PHDI score across 185 countries were ranked. Linear regression models were employed to assess the trend in PHDI score among various subgroups from 1999 to 2018 [35], and to evaluate the association of PHDI score with the incidence, prevalence, mortality, DALYs of CKM diseases, adjusted for year, age, and sex. Restricted cubic spline (3 knots) was used to assess the dose-response relationship between SDI and PHDI score。

In the individual-level analysis using NHANES data, the complex sampling design and sample weights were considered. Participant characteristics were reported as means with standard errors (SE) for continuous variables and as numbers with percentages (%) for categorical variables. Logistic regression models were used to examine the associations between PHDI scores and various CKM diseases and their components. Survival analyses were performed on participants with related diseases to assess the association between PHDI scores and all-cause and CVD mortality. All models were adjusted for NHANES cycles, age, sex, race/ethnicity, education level, marital status, smoking and drinking status, and PA. In the sensitivity analysis, we performed Random Forest imputation for missing data and re-conducted the above analyses to verify the stability of the results [36].

All analyses were conducted using R version 4.4.1 (R Project for Statistical Computing) and Free Statistics software version 2.0. *P* values were adjusted using the Bonferroni method (0.05 divided by the number of diseases).

## 3 Results

### 3.1 Global, regional, and national PHDI score

**Fig. 2:**
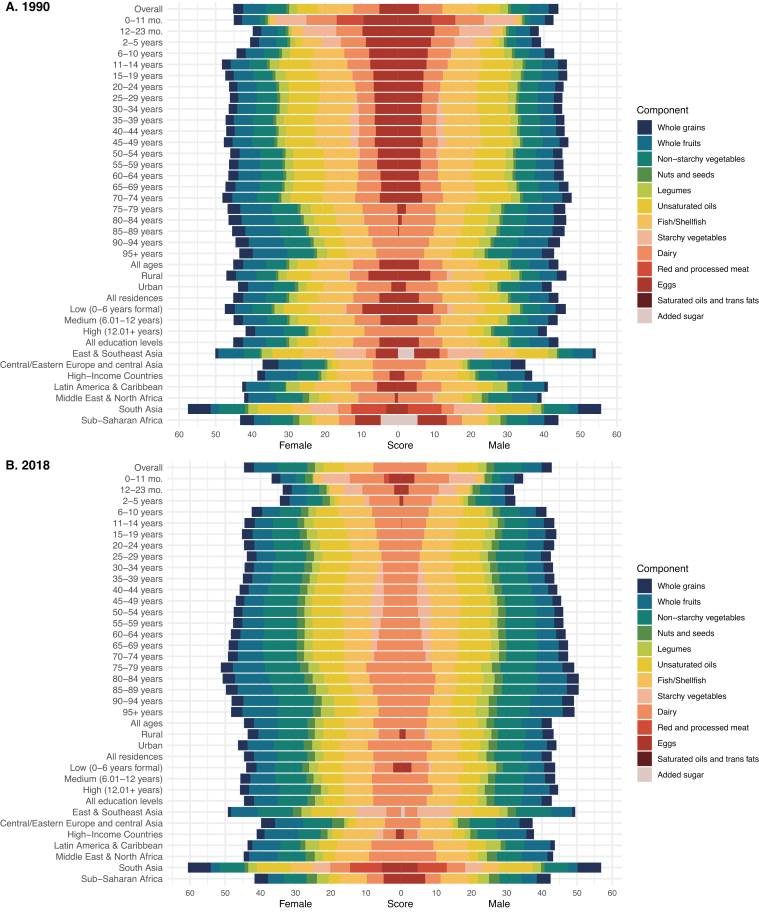
Global mean PHDI component scores by age, sex, education level, residence and region. Abbreviations: PHDI, Planetary Health Diet Index.

Fig. 2 and Supplementary Table S3 illustrated the PHDI scores across different ages, sexes, education levels, residences, and regions from 1990 to 2018.

Between 1990 and 2018, there were no significant changes in PHDI scores for both males and females (*P* for trend > 0.05), with females consistently scoring higher than males. In 2018, the mean PHDI score for males was 42.80 (95% uncertainty interval [UI] 42.49, 46.50), while for females it was 44.65 (95% UI 44.53–47.82). The largest contributors to this difference were whole fruit (female-male, +0.96) and fish/shellfish (female-male, -0.31) (Supplementary Table S5).

From 1990 to 2018, PHDI scores significantly increased among males and females aged ≥75, primarily due to higher scores in nonstarchy vegetables, nuts and seeds (Supplementary Tables S5-S6). In contrast, PHDI scores significantly decreased in individuals under 5 years of age, mainly driven by a decline in the score for eggs. For those under 1 year of age, the score for red/processed meat also significantly decreased (Supplementary Tables S5-S6).

In 2018, the lowest PHDI scores were observed in individuals aged 1 to <2 years, with males scoring 32.05 (95% UI, 28.4–35.28) and females scoring 33.66 (95% UI, 30.38–36.81). Among males, the highest score was in the 80–84 age group, at 50.48 (95% UI, 49.07–49.89), while for females, the highest score was in the 75–79 age group, at 51.23 (95% UI, 49.15–51.02). The greatest positive contributor to the difference between the highest and lowest PHDI scores, regardless of sex, was unsaturated oils, while the largest negative contributor was starchy vegetables (Supplementary Tables S5-S6).

From 1990 to 2018, individuals residing in rural areas or with lower educational levels initially had higher PHDI scores. However, by 2018, those in urban areas or with higher educational levels scored higher. This shift was primarily driven by a narrowing of the gap in egg consumption scores between the two groups (Supplementary Tables S4-S5). Notably, from 1990 to 2018, individuals in urban areas or with higher educational levels consistently had higher scores for dairy consumption.

From 1990 to 2018, PHDI scores significantly increased among males in Central/Eastern Europe and Central Asia, as well as in the Middle East & North Africa. In females, significant increases were observed in Central/Eastern Europe and Central Asia, and High-Income Countries (Supplementary Table S4). In 2018, South Asia had the highest scores, with males scoring 56.84(45.9–50.55) and females scoring 60.59(49.13–53.67). In contrast, Central/Eastern Europe and Central Asia recorded the lowest scores, with males at 37.36(32.97–43.59) and females at 39.8(35.37–46.34). The positive differences were primarily mediated by red/processed meat and unsaturated oils, while the negative differences were attributed to whole fruits, nuts and seeds, and fish/shellfish (Supplementary Table S4-S5).

**Fig. 3:**
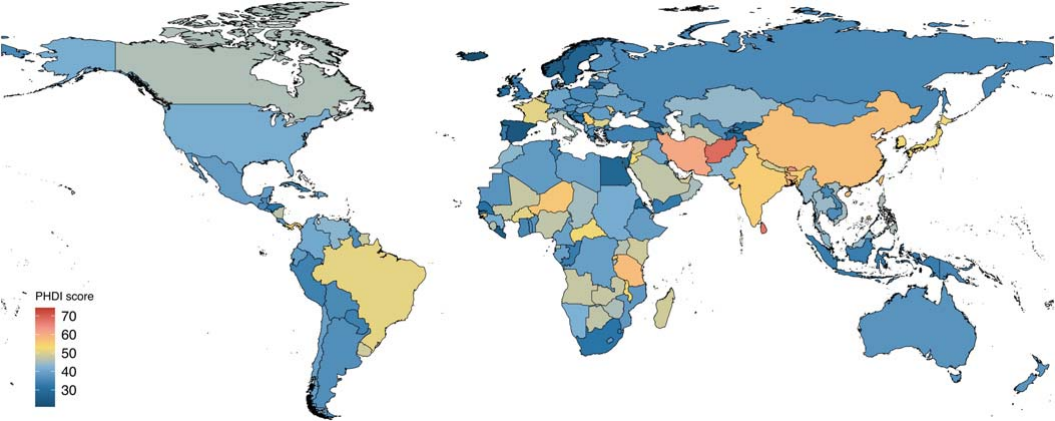
National mean PHDI scores in 2018. Abbreviations: PHDI, Planetary Health Diet Index.

The SDI exhibited a significant negative relationship with PHDI, primarily driven by animal-derived foods such as red/processed meat and eggs (Supplementary Fig. 2). In 2018, the countries with the highest PHDI scores for males were Samoa, Sri Lanka, and Maldives, while for females, the top countries were Samoa, Afghanistan, and Sri Lanka (Fig. 3, Supplementary Table S7).

### 3.2 PHDI and CKM disease

**Fig. 4:**
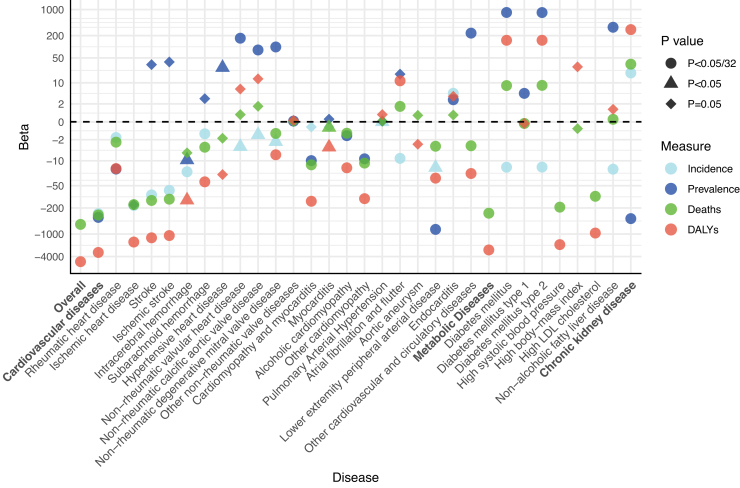
Association between PHDI score and the global burden of CKM diseases. Abbreviations: CKM, cardiovascular, kidney, and metabolic; DALYs, disability-adjusted life years; LDL, low-density lipoprotein; PHDI, Planetary Health Diet Index. All models were adjusted for year, age, and sex.

After adjusting for year, age, and sex, each 1-point increase in PHDI was associated with a decrease in the overall mortality, and DALYs of CKM disease (Fig. 4). However, the associations varied across CVD subtypes; for instance, higher PHDI was associated with lower burdens of rheumatic heart disease, ischemic heart disease, and stroke, while it paradoxically associated with higher burdens of atrial fibrillation/flutter and endocarditis. While PHDI was associated with a decrease in the incidence of DM, it did not reduce its mortality or DALYs. Additionally, an increase in PHDI associated with a higher burden of CKD.

**Fig. 5:**
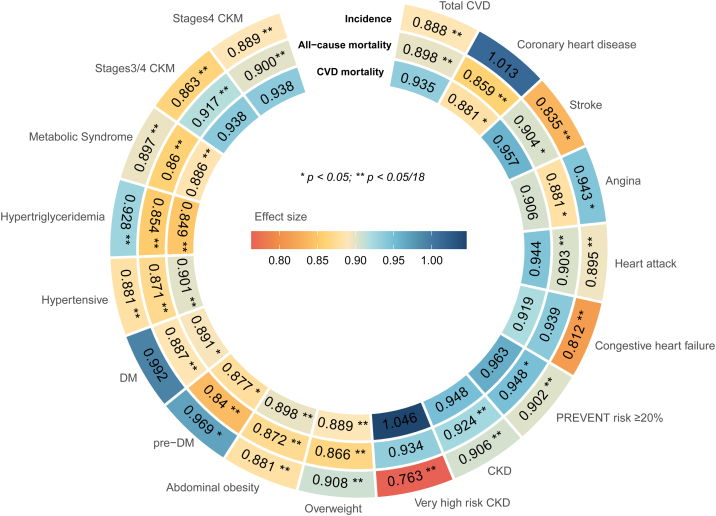
Association between PHDI score and the incidence and mortality of CKM diseases in NHANES. Abbreviations: CKD, chronic kidney disease; CKM, cardiovascular, kidney, and metabolic; CVD, cardiovascular disease; DALYs, disability-adjusted life years; DM, diabetes mellitus; LDL, low-density lipoprotein; NHANES, National Health and Nutrition Examination Survey; PHDI, Planetary Health Diet Index. All models were adjusted for NHANES cycles, age, sex, race/ethnicity, education level, marital status, smoking and drinking status, and physical activity.

In the analysis based on NHANES, we included 45,460 participants with available PHDI and covariates, with a weighted mean age of 47.21 years (SE = 0.19), and 51.40% were female (Supplementary Table S8). Fig. 5 illustrated the association between individual PHDI scores and CKM diseases in NHANES. Higher PHDI scores significantly reduced the incidence of CKM diseases: each 10-point increase was associated with a 13.7% lower risk of developing stage 3/4 CKM syndrome and an 11.1% reduction for stage 4 CKM syndrome. For CVD, a 10-point increase in PHDI was linked to reduced risks of total CVD (11.2%), stroke (16.5%), angina (5.7%), heart attack (10.5%), and congestive heart failure (18.8%), as well as a lower 10-year CVD event risk as predicted by the PREVENT equation. Additionally, higher PHDI scores were associated with reduced risks of all metabolic diseases and a decreased likelihood of very high-risk CKD. PHDI was associated with lower all-cause mortality in individuals with CKM diseases (except for congestive heart failure), and significantly reduced CVD mortality among those with coronary heart disease or metabolic diseases. After imputing the missing data using the Random Forest method, we reanalyzed the data and found consistent results (Supplementary Fig. S3).

## 4 Discussion

From 1990 to 2018, significant changes in global PHDI patterns were observed, such as an significantly increase in PHDI scores among individuals aged ≥75, a decrease in scores among those under 5, and a decline in global egg consumption scores. In 2018, higher PHDI scores were observed among female, older adults, urban residents, and individuals with higher education levels. Notable regional variations in PHDI scores were also found, with a negative association between PHDI and SDI. Globally, higher PHDI scores were linked to a lower burden of overall CKM diseases, although the direction of these associations varied across CKM subcategories. At the individual level, higher PHDI was also found to be associated with lower risks of CKM diseases and related mortality.

Although there were some differences in calculation methods, previous cohort studies from various countries worldwide have reported similar findings, showing that individuals with higher PHDI scores are more likely to be older and female, consistent with our results [[37], [38], [39], [40], [41], [42]]. As the PHDI score increases, the proportion of individuals with higher education levels also rises accordingly [38,39,42]. The GDD, which incorporates representative dietary surveys from around the world, which provided a more comprehensive and high-dimensional estimate of global dietary quality. Therefore, our study further validated and extended previous literature on this topic.

Global egg consumption scores from 1990 to 2018 have shown a decline, particularly among children under five. This trend may highlighted the challenge of balancing environmental sustainability with human health. The EAT-Lancet Commission recommended an intake of about 13 g of eggs per day (1.5 eggs per week) based on a 2500 kcal/day diet [25]. According to the GDD, egg consumption in different age groups increased from 1990 to 2018: for children aged 0 to <1 year (based on a 700 kcal/day standard), from 3.40 g/day to 5.83 g/day; for 1 to <2 years (based on a 1000 kcal/day standard), from 5.28 g/day to 9.04 g/day; and for 2–5 years (based on a 1300 kcal/day standard), from 7.44 g/day to 12.71 g/day. This rise in consumption has contributed to a decline in scores compared to 1990. However, eggs were controversial due to their cholesterol content. Previous studies showed that consuming one egg per day does not significantly increase the risk of CVD [43,44]. In fact, eggs provided essential nutrients and bioactive compounds that benefit adults with chronic diseases and help prevent malnutrition in children [45]. A randomized controlled trial by Iannotti et al. demonstrated that daily consumption of one medium-sized egg (∼50 g) significantly improved stunting and underweight in children aged 6–9 months [46]. Due to their low cost, increased egg consumption may benefit low-income populations with poor dietary quality [25]. In conclusion, while the environmental impact of egg consumption remained a concern, its nutritional benefits made it a valuable food source, particularly for vulnerable populations. It was crucial to balance sustainability with the health advantages eggs offer, especially in regions where they can improve dietary quality and combat malnutrition.

This study highlighted another critical issue: the increasing global consumption of red/processed meat (approximately 72.41 g/day in 1990, rising to 86.78 g/day in 2018, based on a 2500 kcal/day standard), saturated oils/trans fats (14.19% of daily energy intake in 1990, increasing to 14.59% in 2018, based on a 2500 kcal/day standard) and added sugars (10.97% of daily energy intake in 1990, increasing to 14.64% in 2018, based on a 2500 kcal/day standard), despite regional differences (for instance, red/processed meat consumption in South Asia is lower, with 2018 consumption averaging around 12.37 g/day). Consumption in many age groups far exceeds the maximum recommended levels set by the EAT-Lancet Commission (red/processed meat 28 g/day; saturated oils/trans fats, 3.8% of daily energy intake; added sugars, 5% of daily energy intake). Among the various components of the PHDI, red/processed meat had the most significant environmental impact, with implications for energy use, land utilization, and greenhouse gas emissions [25]. Numerous studies linked the consumption of red/processed meat to an increased risk of chronic diseases, including CKM diseases [[47], [48], [49]], and an elevated risk of mortality [50]. Similar associations were observed for saturated oils/trans fats and added sugars [[51], [52], [53], [54], [55]]. The high intake of red/processed meat and saturated oils/trans fats, along with added sugars, presented a significant challenge to both human health and environmental sustainability, underscoring the need for future population-based dietary interventions to reduce consumption and promote a shift toward healthier, more sustainable diets.

Previous studies have explored the association between the PHDI/EAT-Lancet diet and CVD; however, most of these studies were limited to characteristic cohorts, and their conclusions remain controversial. For instance, Berthy et al. conducted a study based on the French adult NutriNet-Santé cohort with 62,382 participants and found no association between PHDI and CVD incidence over a median follow-up of 8.1 years [56]. Similarly, Karavasiloglou et al. found no association between the EAT-Lancet diet and CVD events in a prospective cohort of over 400,000 individuals from the UK Biobank [57]. However, the limitation of this study was that the EAT-Lancet diet was measured only once at baseline, and its calculation relied on a 29-item food intake questionnaire, potentially leading to misestimation of PHDI. In contrast, Sotos-Prieto et al. focused on participants who completed at least two 24-hour dietary interviews and found that the mean PHDI from multiple measurements was associated with a reduced risk of CVD, including myocardial infarction and stroke [14]. Additionally, Sawicki et al. conducted a pooled analysis of three American cohorts, Nurses’ Health Study I, Nurses’ Health Study II, and Health Professionals Follow-up Study, and reported that adherence to a higher PHDI was associated with a reduced risk of CVD, including coronary heart disease, stroke, and ischemic stroke [2]. Furthermore, some studies have investigated the relationship between PHDI and specific CVD subtypes. Zhang et al. found that adherence to a higher-quality EAT-Lancet diet was associated with lower incidences of atrial fibrillation and heart failure based on two studies [13,58].

Several studies also explored the association between the PHDI and cardiometabolic risk factors. A studies from Brazil found that adherence to the EAT-Lancet diet was associated with lower levels of blood pressure, total cholesterol, LDL cholesterol, and non-HDL cholesterol [59]. Furthermore, the EAT-Lancet diet had been linked to a reduced risk of obesity, type 2 DM, and metabolic dysfunction-associated steatotic liver disease [38,60,61]. Additionally, research by Teixeira et al. further confirmed that adherence to the EAT-Lancet diet from an early age may help reduce cardiometabolic risk during early adolescence [15].

In this study, we found that, from a global perspective, higher PHDI was associated with lower incidence, prevalence, mortality, and DALYs related to overall CKM. However, the burden of certain CKM subtypes, such as atrial fibrillation/flutter and endocarditis, and CKD appeared to be increasing, presenting an unexpected conflict with our individual-level analysis and previous studies. This discrepancy may stem from potential biases inherent in the ecological study framework. Since ecological studies used populations as the unit of observation and analysis, they could not establish individual-level exposure-disease relationships. The data reflected average levels at the group level, providing a coarse description that prevents causal inferences between exposure and disease. Additionally, confounding factors may lead to discrepancies between study results and actual conditions. Nonetheless, macro-level analyses offered research hypotheses and clues for population-based interventions, underscoring the importance of further investigating different CKM subtypes. On the other hand, as Milner et al. pointed out, the impact of sustainable dietary patterns on health and environmental sustainability may depend critically on local contextual factors [27]. While numerous studies had explored the impact of the PHDI on individual health, remained a need for more tailored and rigorous research to understand its broader macro-level effects on human health.

Potential limitations should be acknowledged. First, due to inherent methodological constraints in the GDD and GBD study, data quality varied depending on the national sources. Data collection and entry practices may differ across countries based on factors such as population size and economic conditions. In particular, low-income countries with limited data availability may suffer from incomplete or poor-quality data, potentially leading to inaccurate estimates [19,21,62]. Second, the GDD did not include data on poultry and provide a detailed breakdown of nonsoy legumes and soybean/soy foods, which may introduce bias in the calculation of the PHDI. Additionally, the PHDI was based on a standard of 2500 kcal/day for adults. In the GDD data, different caloric requirements were standardized for different age groups. Therefore, in our study, when comparing scores across different age groups, we standardized each stratum to 2500 kcal/day, following the approach used by Miller et al. [26], to ensure comparability of the scores. However, applying the same PHDI calculation standards across different age groups may be inappropriate, highlighting the need for further research on this issue. Third, both global and individual-level analyses may still have been subject to potential confounding factors. Fourth, in individual-level analyses, some CKM indicators were derived from self-reported data, which may introduce recall bias and result in classification errors. Fifth, given the observational design of NHANES, any causal inferences regarding the incidence of CKM diseases should be made with caution. Finally, the application of the findings from this study should be approached carefully, as the impact of sustainable dietary patterns on health and environmental sustainability may depend critically on local contextual factors [27].

## 5 Conclusion

Between 1990 and 2018, the patterns of the PHDI underwent significant changes. PHDI scores vary according to demographic characteristics, geographical regions, and socioeconomic development, with animal-based foods, such as red and processed meat, potentially serving as important driving factors. This study supports the conclusion that adherence to a higher PHDI may help reduce the burden of CKM diseases, including both disease incidence and long-term prognosis. However, further research is necessary to investigate the subtypes of CKM diseases.

## Authorship contribution statement

Conceptualization: H.T., X.Z., Y.C.; Data Management and Analysis: H.T., X.Z.; Figure Creation: H.T., N.L., J.H.; Writing - Original Draft Preparation: H.T., N.L., J.H., Q.Y., H.L., M.L.; Writing - Review and Editing: S.W., J.W., H.L., J.H., P.C., L.J.; Provided Critical Revisions to the Manuscript: X.T., Y.C.; Project Management: X.T., Y.C.

## Ethics statement

The NHANES protocol was reviewed and approved by the by the Institutional Review Board of the National Center for Health Statistics, with all participants providing written informed consent. As the current study utilized publicly available deidentified data, it was exempted from the requirement to obtain informed consent in accordance with the relevant regulations of the Shantou University Medical College Institutional Review Board.

## Role of the funding source

The funders of the study had no role in study design, data collection, data analysis, data interpretation, or writing of the report. All authors had full access to all the data in the study and accepted responsibility to submit for publication.

## Funding sources

This research was supported by 2020 Li Ka Shing Foundation Cross-Disciplinary Research Grant [2020LKSFG19B], “Dengfeng Project” for the construction of high-level hospitals in Guangdong Province - the First Affiliated Hospital of Shantou University Medical College [2020], Science and Technology project in Guangdong Province [2021010303], 2021 Guangdong Province Science and Technology Special Fund [2021-88-53], 2022 Guangdong Province Science and Technology Special Fund [2022-124-6], National Health Commission Medical and Health Technology Development Research Center [WKZX2022JG0138], and Innovation Team Project of Guangdong Ordinary Colleges and Universities (Natural Science) [2024KCXTD019].

## Declaration of Generative AI and AI-assisted technologies in the writing process

During the preparation of this work the authors used ChatGPT in order to improve language and readability. The authors reviewed and edited the content and took full responsibility for the content of the publication.

## Data availability

The data utilized in this study are publicly available from the following sources: the Global Dietary Database (GDD) at https://globaldietarydatabase.org/; the Global Burden of Disease (GBD) data used in the analyses can be accessed at https://ghdx.healthdata.org/gbd-results-tool; the National Health and Nutrition Examination Survey (NHANES) at https://www.cdc.gov/nchs/nhanes/index.htm.

## Declaration of competing interest

The authors declare no competing interests that pertain to this work.
