# Supplementary material for: Temporal trends in the planetary health diet index and its association with cardiovascular, kidney, and metabolic diseases: A comprehensive analysis from global and individual perspectives
Source: J Nutr Health Aging. 2025 Feb 21;29(5):100520. doi: 10.1016/j.jnha.2025.100520 (PMC12180045; doi:10.1016/j.jnha.2025.100520)
Supplement: Supplementary file 1 [file mmc1.docx]

**Supplementary Materials Files**

To: **Temporal Trends in the Planetary Health Diet Index and Its Association with Cardiovascular, Kidney, and Metabolic Diseases: A Comprehensive Analysis from Global and Individual Perspectives**

Haoxian Tang, Xuan Zhang, Nan Luo, Jingtao Huang, Qinglong Yang, Hanyuan Lin, Mengyue Lin, Shiwan Wu, Jiasheng Wen, Guinan Hong, Pan Chen, Liwen Jiang, Yequn Chen Xuerui Tan

**Supplementary Figure**

Supplementary Figure 1. National mean Planetary Health Diet Index (PHDI) scores by sex, in 2018.

Supplementary Figure 2. Association of socio-demographic index with planetary health diet index score and its components by restricted cubic splines regression.

Supplementary Figure 3. Association between PHDI score and the incidence and mortality of CKM diseases in NHANES, with missing values imputed using the random forest estimation method.

**Supplementary Table**

Supplementary Table 1. Calculation of the planetary health diet index, for an intake of 2500 kcal/day

Supplementary Table 2. Definitions of CKM Health Stages (Green indicates that all criteria must be met, yellow indicates that meeting any one criterion is sufficient)

Supplementary Table 3. Algebra for 10-Year Equations Calculation by PREVENT models

Supplementary Table 4. Planetary health diet index score by age, education, residence, and region in 1990-2018.

Supplementary Table 5. Absolute differences in PHDI scores between the highest and lowest subgroups of each demographic characteristic

Supplementary Table 6. Absolute differences in PHDI scores between 2018 and 1990 of each age groups.

Supplementary Table 7. Planetary health diet index score and rankings for 185 countries in 2018.

Supplementary Table 8. Characteristics of Participants in the National Health and Nutrition Examination Survey

**Supplementary Figure 1. National mean Planetary Health Diet Index (PHDI) scores by sex, in 2018.**


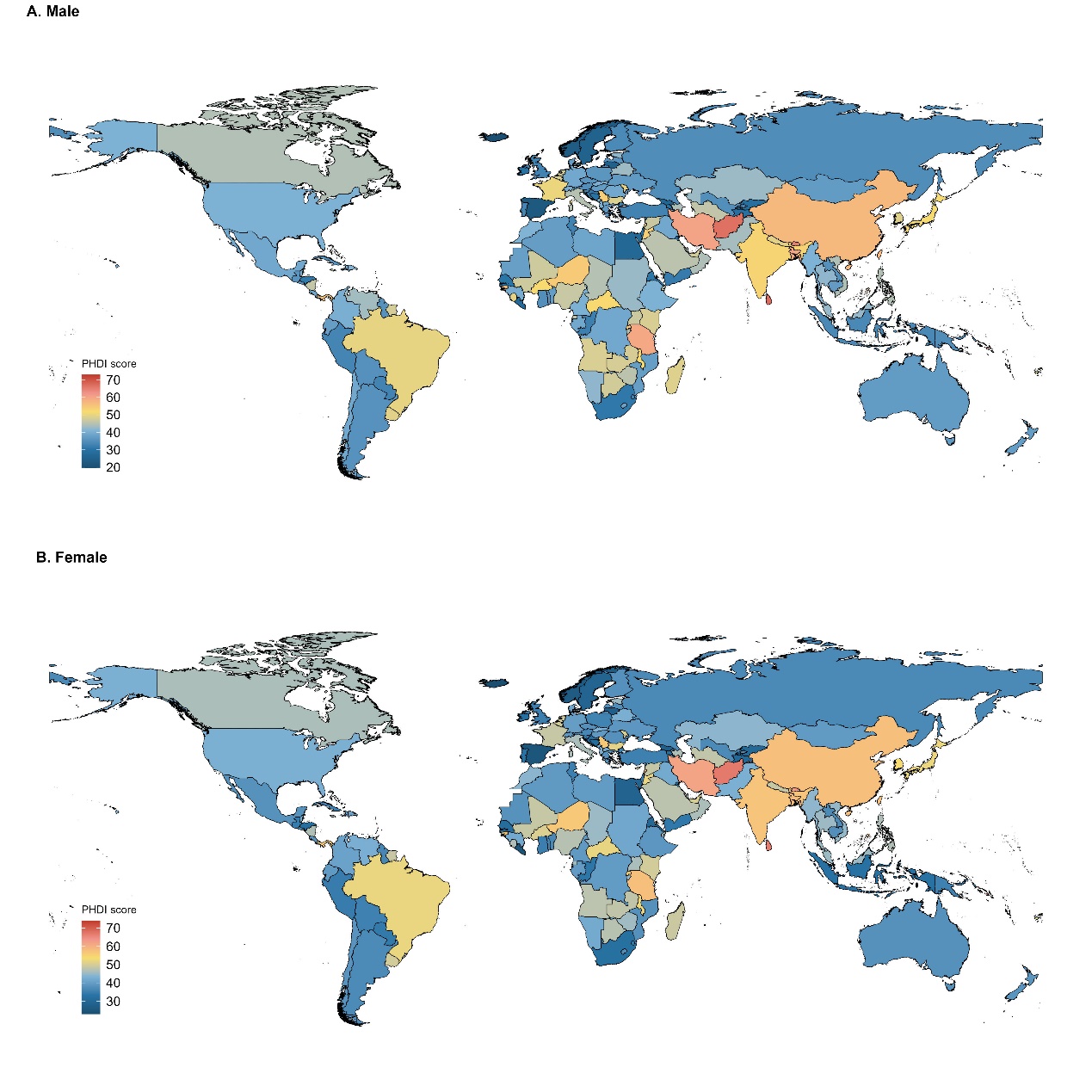


**Supplementary Figure 2. Association of socio-demographic index with planetary health diet index score and its components by restricted cubic splines regression.**


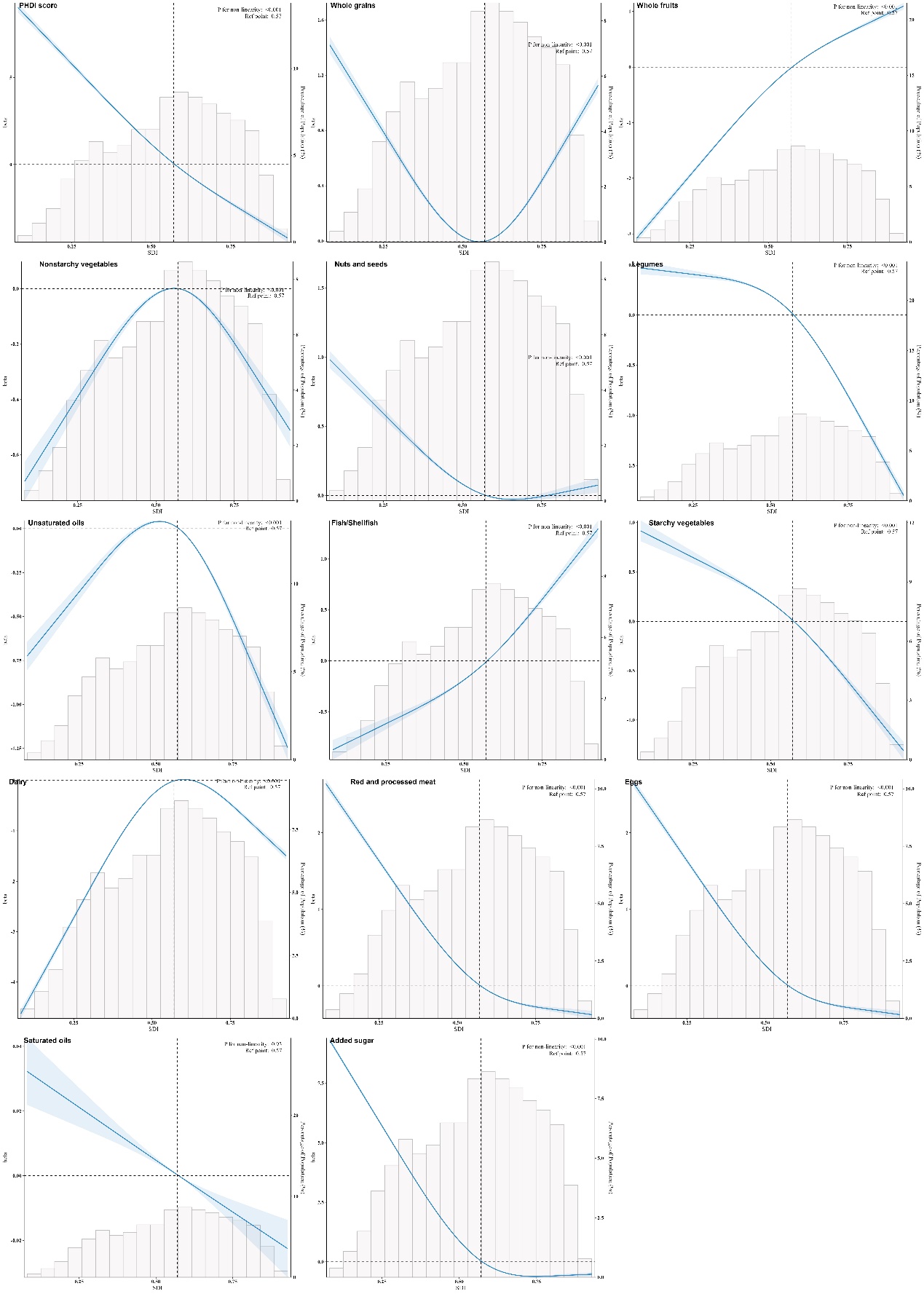


Include data from 1990-2018, model was adjusted for age, sex and year.

**Supplementary Figure 3. Association between PHDI score and the incidence and mortality of CKM diseases in NHANES, with missing values imputed using the random forest estimation method.**


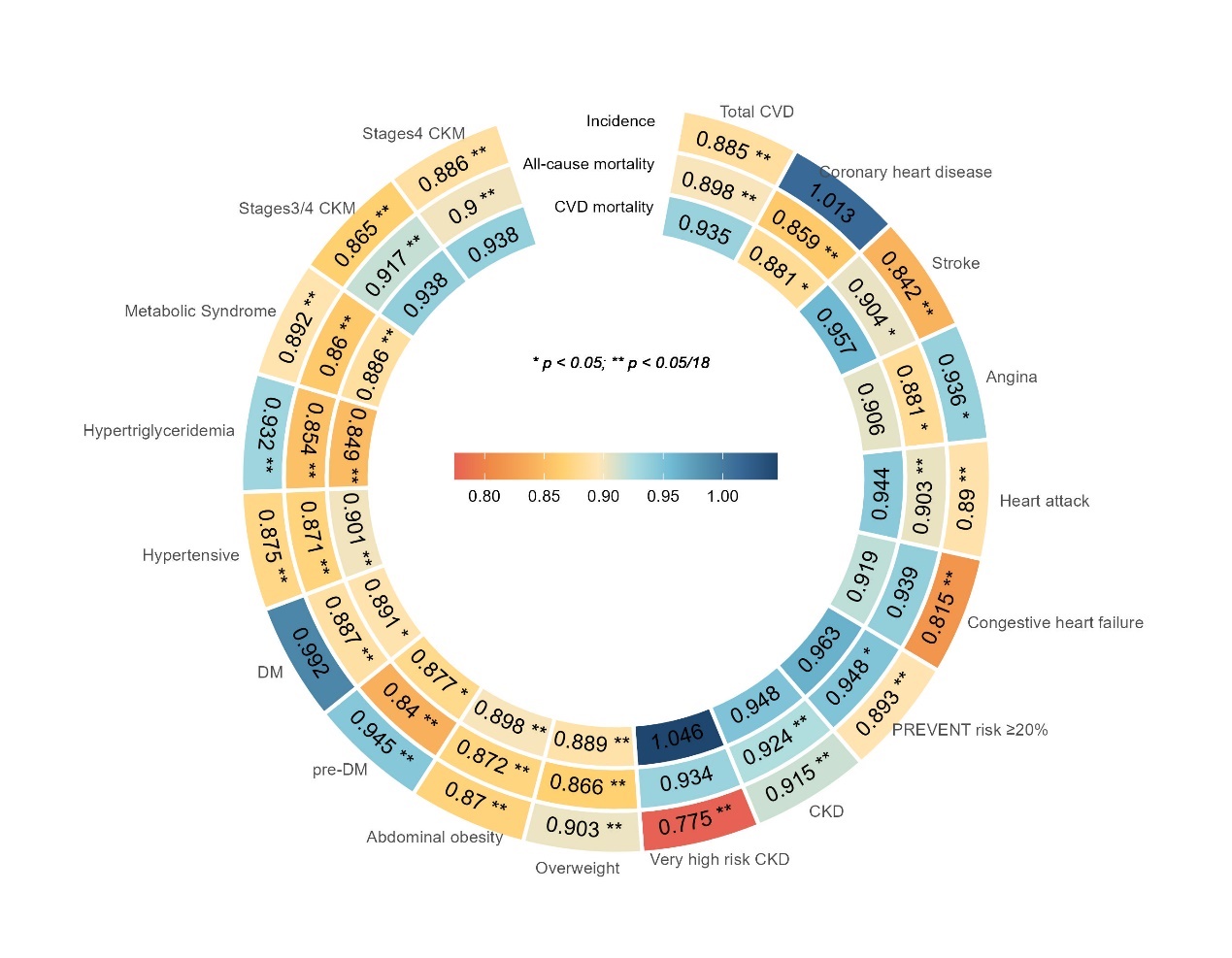


Abbreviations: CKD, chronic kidney disease; CKM, cardiovascular, kidney, and metabolic; CVD, cardiovascular disease; DALYs, disability-adjusted life years; DM, diabetes mellitus; LDL, low-density lipoprotein; NHANES, National Health and Nutrition Examination Survey; PHDI, Planetary Health Diet Index. All models were adjusted for NHANES cycles, age, sex, race/ethnicity, education level, marital status, smoking and drinking status, and physical activity.

**Supplementary Table 1. Calculation of the planetary health diet index, for an intake of 2500 kcal/day**

|  | **Dietary component** | **EAT-Lancet recommendations** | | **0 points** | **0-10 points** | **10 points** | **10-0 points** | **0 points** |
| --- | --- | --- | --- | --- | --- | --- | --- | --- |
|  |  | **g/day** | **kcal/day** |  |  |  |  |  |
|  | **Adequacy components** |  |  |  |  |  |  |  |
| 1 | Whole grains | 232(0-464g) | 811 | 0g | 0-232g | 232g | 232-464g | ≥464 g |
| 2 | Whole fruits | 200(100-300g) | 126 | 0g | 0-200g | ≥200g |  |  |
| 3 | Nonstarchy vegetables | 300(200-600g) | 78 | 0g | 0-300g | ≥300g |  |  |
| 4 | Nuts and seeds | 50(0-75g) | 291 | 0g | 0-50g | ≥50g |  |  |
|  | Legumes |  |  |  |  |  |  |  |
| 5 | Nonsoy legumes (e.g., dry beans, lentils, peas) | 50(0-100g) | 172 | 0g | 0-100g | ≥100g |  |  |
| 6 | Soybean/ soy foods (dry weight) | 25(0-50g) | 112 | 0g | 0-50g | ≥50g |  |  |
| 7 | Unsaturated oils | 40(20-80g) | 354 | 0g (or 0% of total energy intake) | 0-40g (0-14% of total energy intake) | 40g (or 14 % of total energy intake) | 40-80g (or 28% of total energy intake) | 80g (or 28% of total energy intake) |
| 8 | Fish/Shellfish | 28(0-100g) | 40 | 0g | 0-28 | 28g | 28-100g | ≥100g |
|  | **Moderation components** |  |  |  |  |  |  |  |
| 9 | Starchy vegetables | 50(0-100g) | 39 | 0g | 0-50g | 50 | 50-100g | ≥100g |
| 10 | Dairy | 250(0-500g) | 153 | 0g | 0-250g | 250g | 250-500g | ≥500g |
| 11 | Red and processed meat | 14(0-28g) | 30 | 0g | 0-14g | 14g | 14-28g | ≥28g |
| 12 | Poultry | 29(0-58g) | 62 | 0g | 0-29g | 29g | 29-58g | ≥58g |
| 13 | Eggs | 13(0-25g) | 19 | 0g | 0-13g | 13g | 13-25g | ≥25g |
| 14 | Saturated oils and trans fats | 11.8(0-11.8g) | 96 |  |  | 0g (or 0% of total energy intake) | 0-11.8g (or 0-3.8% of total energy intake) | ≥11.8g (or ≥3.8% of total energy intake) |
| 15 | Added sugar | 31(0-31g) | 120 |  |  | 0g (or 0% of total energy intake) | 0-31g (or 0-5% of total energy intake) | ≥31g (or ≥5% of total energy intake) |

The total score for legumes is calculated by taking the scores of Nonsoy legumes and Soybean/Soy foods, each multiplied by 0.5 and then summed together. In the Global Dietary Database, the aggregate value for legumes is provided without further classification. For this analysis, we define a perfect score of 10 points as equivalent to an intake of 150 grams.

**References:**

1. Chen H, Wang X, Ji JS, et al. Plant-based and planetary-health diets, environmental burden, and risk of mortality: a prospective cohort study of middle-aged and older adults in China. *Lancet Planet Health*. 2024;8(8):e545-e553. doi:10.1016/S2542-5196(24)00143-8

2. Sawicki CM, Ramesh G, Bui L, et al. Planetary health diet and cardiovascular disease: results from three large prospective cohort studies in the USA. Lancet Planet Health. 2024;8(9):e666-e674. doi:10.1016/S2542-5196(24)00170-0

3. Willett W, Rockström J, Loken B, et al. Food in the Anthropocene: the EAT-Lancet Commission on healthy diets from sustainable food systems [published correction appears in Lancet. 2019 Feb 9;393(10171):530. doi: 10.1016/S0140-6736(19)30212-0] [published correction appears in Lancet. 2019 Jun 29;393(10191):2590. doi: 10.1016/S0140-6736(19)31428-X] [published correction appears in Lancet. 2020 Feb 1;395(10221):338. doi: 10.1016/S0140-6736(20)30144-6] [published correction appears in Lancet. 2020 Oct 3;396(10256):e56. doi: 10.1016/S0140-6736(20)31828-6]. Lancet. 2019;393(10170):447-492. doi:10.1016/S0140-6736(18)31788-4

**Supplementary Table 2. Definitions of CKM Health Stages (Green indicates that all criteria must be met, yellow indicates that meeting any one criterion is sufficient)**

| **CKM health stages** | **Definition** | **Indicators** | **Criterion** | **Condition** |
| --- | --- | --- | --- | --- |
| Stage 0: No CKM health risk factors | Individuals without overweight/obesity, metabolic risk factors (hypertension, hypertriglyceridemia, MetS, diabetes), CKD, or subclinical/clinical CVD | Without overweight/obesity | BMI <25 kg/m^2^ (or <23 kg/m^2^ if Asian ancestry) | All criteria are met |
|  |  | Without abdominal obesity | Waist circumference <88/102 cm in female/male (or if Asian ancestry <80/90 cm in female/male) |  |
|  |  | Without prediabetes/diabetes | Fasting blood glucose <100 mg/dL and HbA1c < 5.7% and without self-reported diagnosis of prediabetes/diabetes, use of insulin, or oral hypoglycemic agents |  |
|  |  | Without hypertension | SBP <130 mmHg and DBP <80 mmHg without self-reported diagnosis of hypertension or use of antihypertensive medications |  |
|  |  | Without hypertriglyceridemia | Triglycerides < 135 mg/dL |  |
|  |  | Without CKD | Low-risk CKD in KDIGO classification according to eGFR and  UACR: UACR < 30 mg/g and eGFR ≥ 60 ml/min/1.73m^2^. |  |
|  |  | Without subclinical/clinical CVD | Predicted 10-year CVD risk < 20% |  |
|  |  |  | Without history of coronary heart failure, coronary heart disease, heart attack, or stroke |  |
| Stage 1: Excess and/or dysfunctional adiposity | Individuals with overweight/obesity, abdominal obesity, or dysfunctional adipose tissue, without the presence of other metabolic risk factors or CKD | With overweight/obesity | BMI ≥25 kg/m^2^ (or ≥23 kg/m^2^ if Asian ancestry) | Any of the three criteria is met |
|  |  | With abdominal obesity | Waist circumference ≥88/102 cm in women/men (or if Asian ancestry, ≥80/90 cm in women/men) |  |
|  |  | Prediabetes | Fasting blood glucose ≥100-124 mg/dL or HbA1c between 5.7% and 6.4% |  |
|  |  | Without hypertension | SBP <130 mmHg and DBP <80 mmHg without self-reported diagnosis of hypertension or use of antihypertensive medications | All criteria are met |
|  |  | Without hypertriglyceridemia | Triglycerides < 135 mg/dL |  |
|  |  | Without CKD | Low-risk CKD in KDIGO classification according to eGFR and  UACR: UACR < 30 mg/g and eGFR ≥ 60 ml/min/1.73m^2^. |  |
|  |  | Without subclinical/clinical CVD | Predicted 10-year CVD risk < 20% |  |
|  |  |  | Without history of coronary heart failure, coronary heart disease, heart attack, or stroke |  |
| Stage 2: Metabolic risk factors and CKD | Individuals with metabolic risk factors (hypertriglyceridemia, hypertension, MetS, diabetes) or CKD | With hypertriglyceridemia | Triglycerides ≥ 135 mg/dL | Any of the five criteria is met |
|  |  | With hypertension | SBP ≥130mm Hg or DBP ≥80mm Hg or self-reported diagnosis of hypertension or use of antihypertensive medication |  |
|  |  | With diabetes | Fasting blood glucose ≥ 125 mg/dL or HbA1c ≥ 6.5% or self-reported diagnosis of diabetes, use of insulin, or oral hypoglycemic agents |  |
|  |  | With MetS | MetS is defined by the presence of ≥3 of the following: (1) waist circumference ≥88 cm for women and ≥102 cm for men (if Asian ancestry, ≥80 cm for women and ≥90 cm for men), (2) high-density cholesterol <40 mg/dL for men and <50 mg/dL for women; (3) triglycerides ≥150 mg/dL; (4) elevated blood pressure (systolic blood pressure ≥130 mm Hg and/or diastolic blood pressure ≥80 mm Hg and/or use of antihypertensive medications); and (5) fasting blood glucose ≥100 mg/dL. |  |
|  |  | With moderate-to-high-risk CKD | Moderate-to-high-risk CKD in KDIGO classification: UACR ≥ 30 mg/g and eGFR ≥ 60 ml/min/1.73m^2^, UACR < 300 mg/g and eGFR 45-59 ml/min/1.73m^2^, or UACR < 30 mg/g and eGFR 30-44 ml/min/1.73m^2^. |  |
|  |  | Without very high-risk CKD | No very high-risk CKD in KDIGO classification: eGFR ≥ 60 ml/min/1.73m^2^, eGFR 45-59 ml/min/1.73m^2^ and UACR <300 mg/g, eGFR30 ml/min/1.73m^2^, eGFR 30-44 ml/min/1.73m^2^ and UACR <30 mg/g | All criteria are met |
|  |  | Without subclinical/clinical CVD | Predicted 10-year CVD risk < 20% |  |
|  |  |  | Without history of coronary heart failure, coronary heart disease, heart attack, or stroke |  |
| Stage 3: Subclinical CVD in CKM | Subclinical CVD in CKM | With very high-risk CKD | Very high-risk CKD in KDIGO classification: eGFR＜30 ml/min/1.73m^2^, eGFR 30-44 ml/min/1.73m^2^ and UACR ≥30 mg/g, or eGFR 45-59 ml/min/1.73m^2^ and UACR ≥300 mg/g | Any of the two criteria is met |
|  |  | With subclinical CVD | Predicted 10-y CVD risk ≥ 20% |  |
|  |  | Without clinical CVD | Without history of coronary heart failure, coronary heart disease, heart attack, or stroke | The criterion is met |
|  |  | With overweight/obesity | BMI ≥25 kg/m^2^ (or ≥23 kg/m^2^ if Asian ancestry) | Any of the eight criteria is met |
|  |  | With abdominal obesity | Waist circumference ≥88/102 cm in women/men (or if Asian ancestry, ≥80/90 cm in women/men) |  |
|  |  | With hypertriglyceridemia | Triglycerides ≥ 135 mg/dL |  |
|  |  | With hypertension | SBP ≥130mm Hg or DBP ≥80mm Hg or self-reported diagnosis of hypertension or use of antihypertensive medication |  |
|  |  | With prediabetes | Fasting blood glucose ≥100-124 mg/dL or HbA1c between 5.7% and 6.4% |  |
|  |  | With diabetes | Fasting blood glucose ≥ 125 mg/dL or HbA1c ≥ 6.5% or self-reported diagnosis of diabetes, use of insulin, or oral hypoglycemic agents |  |
|  |  | With MetS | MetS is defined by the presence of ≥3 of the following: (1) waist circumference ≥88 cm for women and ≥102 cm for men (if Asian ancestry, ≥80 cm for women and ≥90 cm for men), (2) high-density cholesterol <40 mg/dL for men and <50 mg/dL for women; (3) triglycerides ≥150 mg/dL; (4) elevated blood pressure (systolic blood pressure ≥130 mm Hg and/or diastolic blood pressure ≥80 mm Hg and/or use of antihypertensive medications); and (5) fasting blood glucose ≥100 mg/dL. |  |
|  |  | With moderate-to-high-risk CKD | Moderate-to-high-risk CKD in KDIGO classification: UACR ≥ 30 mg/g and eGFR ≥ 60 ml/min/1.73m^2^, UACR < 300 mg/g and eGFR 45-59 ml/min/1.73m^2^, or UACR < 30 mg/g and eGFR 30-44 ml/min/1.73m^2^. |  |
| Stage 4: Clinical CVD in CKM | Clinical CVD (coronary heart disease, heart failure, stroke, peripheral artery disease, AFib) among individuals with excess/dysfunctional adiposity, other metabolic risk factors, or CKD | With clinical CVD | History of coronary heart failure, coronary heart disease, heart attack, or stroke | The criterion is met |
|  |  | With overweight/obesity | BMI ≥25 kg/m^2^ (or ≥23 kg/m^2^ if Asian ancestry) | Any of the nine criteria is met |
|  |  | With abdominal obesity | Waist circumference ≥88/102 cm in women/men (or if Asian ancestry, ≥80/90 cm in women/men) |  |
|  |  | With hypertriglyceridemia | Triglycerides ≥ 135 mg/dL |  |
|  |  | With hypertension | SBP ≥130mm Hg or DBP ≥80mm Hg or self-reported diagnosis of hypertension or use of antihypertensive medication |  |
|  |  | With prediabetes | Fasting blood glucose ≥100-124 mg/dL or HbA1c between 5.7% and 6.4% |  |
|  |  | With diabetes | Fasting blood glucose ≥ 125 mg/dL or HbA1c ≥ 6.5% or self-reported diagnosis of diabetes, use of insulin, or oral hypoglycemic agents |  |
|  |  | With MetS | MetS is defined by the presence of ≥3 of the following: (1) waist circumference ≥88 cm for women and ≥102 cm for men (if Asian ancestry, ≥80 cm for women and ≥90 cm for men), (2) high-density cholesterol <40 mg/dL for men and <50 mg/dL for women; (3) triglycerides ≥150 mg/dL; (4) elevated blood pressure (systolic blood pressure ≥130 mm Hg and/or diastolic blood pressure ≥80 mm Hg and/or use of antihypertensive medications); and (5) fasting blood glucose ≥100 mg/dL. |  |
|  |  | With moderate-to-high-risk CKD | Moderate-to-high-risk CKD in KDIGO classification: UACR ≥ 30 mg/g and eGFR ≥ 60 ml/min/1.73m^2^, UACR < 300 mg/g and eGFR 45-59 ml/min/1.73m^2^, or UACR < 30 mg/g and eGFR 30-44 ml/min/1.73m^2^. |  |
|  |  | With very high-risk CKD | Very high-risk CKD in KDIGO classification: eGFR＜30 ml/min/1.73m^2^, eGFR 30-44 ml/min/1.73m^2^ and UACR ≥30 mg/g, or eGFR 45-59 ml/min/1.73m^2^ and UACR ≥300 mg/g |  |

Abbreviations: BMI, body mass index; CKD, chronic kidney disease; CKM, cardiovascular-kidney-metabolic; CVD, cardiovascular disease; DBP, diastolic blood pressure; eGFR, estimated glomerular filtration rate; MetS, metabolic syndrome; HDL, high-density lipoprotein; KDIGO, The Kidney Disease, Improving Global Outcomes; SBP, systolic blood pressure; UACR, urinary albumin to creatinine ratio.

**References:**

1. Ndumele CE, Rangaswami J, Chow SL, et al. Cardiovascular-Kidney-Metabolic Health: A Presidential Advisory From the American Heart Association [published correction appears in Circulation. 2024 Mar 26;149(13):e1023. doi: 10.1161/CIR.0000000000001241]. Circulation. 2023;148(20):1606-1635. doi:10.1161/CIR.0000000000001184
2. Li J, Lei L, Wang W, et al. Social Risk Profile and Cardiovascular-Kidney-Metabolic Syndrome in US Adults. J Am Heart Assoc. 2024;13(16):e034996. doi:10.1161/JAHA.124.034996

**Supplementary Table 3. Algebra for 10-Year Equations Calculation by PREVENT models**

| **Total CVD (Base 10 year)** | |
| --- | --- |
| **Women** | **log-Odds** = -3.307728 + 0.7939329 × (age – 55) /10 + 0.0305239 × ((TC – HDL) × 0.02586 – 3.5) – 0.1606857 × (HDL × 0.02586 – 1.3) /0.3 – 0.2394003 × (min(SBP, 110) – 110) /20 + 0.360078 × (max(SBP, 110) – 130) /20 + 0.8667604 × (if diabetes) + 0.5360739 × (if current smoker) + 0.6045917 × (min(eGFR, 60) – 60) / -15 + 0.0433769 × (max(eGFR, 60) – 90) / -15 + 0.3151672 × (if using anti-hypertensive medication) – 0.1477655 × (if using statin) – 0.0663612 × (if using anti-hypertensive medication) × (max(SBP, 110) – 130) /20 + 0.1197879 × (if using statin) × ((TC – HDL) × 0.02586 – 3.5) – 0.0819715 × (age – 55) /10 × ((TC – HDL) × 0.02586 – 3.5) + 0.0306769 × (age – 55) /10 × (HDL × 0.02586 – 1.3) /0.3 – 0.0946348 × (age – 55) /10 × (max(SBP, 110) – 130) /20 – 0.27057 × (age – 55) /10 × (if diabetes) – 0.078715 × (age – 55) /10 × (if current smoker) – 0.1637806 × (age – 55) /10 × (min(eGFR, 60) – 60) / -15  **Risk** = exp(log-Odds) / (1 + exp(log-Odds)) |
| **Men** | **log-Odds** = -3.031168 + 0.7688528 × (age – 55) /10 + 0.0736174 × ((TC – HDL) × 0.02586 – 3.5) – 0.0954431 × (HDL × 0.02586 – 1.3) /0.3 – 0.4347345 × (min(SBP, 110) – 110) /20 + 0.3362658 × (max(SBP, 110) – 130) /20 + 0.7692857 × (if diabetes) + 0.4386871 × (if current smoker) + 0.5378979 × (min(eGFR, 60) – 60) / -15 + 0.0164827 × (max(eGFR, 60) – 90) / -15 + 0.288879 × (if using anti-hypertensive medication) – 0.1337349 × (if using statin) – 0.0475924 × (if using anti-hypertensive medication) × (max(SBP, 110) – 130) /20 + 0.150273 × (if using statin) × ((TC – HDL) × 0.02586 – 3.5) – 0.0517874 × (age – 55) /10 × ((TC – HDL) × 0.02586 – 3.5) + 0.0191169 × (age – 55) /10 × (HDL × 0.02586 – 1.3) /0.3 – 0.1049477 × (age – 55) /10 × (max(SBP, 110) – 130) /20 – 0.2251948 × (age – 55) /10 × (if diabetes) – 0.0895067 × (age – 55) /10 × (if current smoker) – 0.1543702 × (age – 55) /10 × (min(eGFR, 60) – 60) / -15  **Risk** = exp(log-Odds) / (1 + exp(log-Odds)) |

**References:**

1. Khan SS, Matsushita K, Sang Y, et al. Development and Validation of the American Heart Association's PREVENT Equations [published correction appears in Circulation. 2024 Mar 12;149(11):e956. doi: 10.1161/CIR.0000000000001230]. Circulation. 2024;149(6):430-449. doi:10.1161/CIRCULATIONAHA.123.067626Novel
2. Khan SS, Coresh J, Pencina MJ, et al. Novel Prediction Equations for Absolute Risk Assessment of Total Cardiovascular Disease Incorporating Cardiovascular-Kidney-Metabolic Health: A Scientific Statement From the American Heart Association. Circulation. 2023;148(24):1982-2004. doi:10.1161/CIR.0000000000001191
3. Li J, Lei L, Wang W, et al. Social Risk Profile and Cardiovascular-Kidney-Metabolic Syndrome in US Adults. J Am Heart Assoc. 2024;13(16):e034996. doi:10.1161/JAHA.124.03499

**Supplementary Table 4.** **Planetary health diet index score by age, education, residence, and region in 1990-2018.**

| **Group** | **1990** | **1995** | **2000** | **2005** | **2010** | **2015** | **2018** | **Coefficient** | **P for trend** |
| --- | --- | --- | --- | --- | --- | --- | --- | --- | --- |
| **Male** |  |  |  |  |  |  |  |  |  |
| Overall | 43.98(41.51, 46.45) | 42.9(39.2, 46.56) | 42.37(40.04, 46.2) | 42.02(40.99, 47.14) | 42.49(42.19, 46.79) | 42.14(41.82, 46.18) | 42.8(42.49, 46.5) | -0.039 | 0.147 |
| 0-11 mo. | 42.63(39.63, 41.02) | 43.55(37.54, 43.17) | 43.05(36.44, 44.41) | 42.69(34.65, 44.26) | 37.25(29.6, 43.46) | 35.49(28.22, 41.71) | 34.66(27.83, 40.91) | **-0.34** | **0.006** |
| 12-23 mo. | 38.59(36.06, 38.64) | 37.67(34.32, 40.43) | 36.88(33.23, 39.89) | 36.61(32.54, 38.71) | 34.54(30.42, 37.19) | 32.21(27.81, 35.41) | 32.05(28.4, 35.28) | **-0.245** | **<0.001** |
| 2-5 years | 39.2(33.96, 42.33) | 38.21(32.33, 42.37) | 37.68(31.47, 42.31) | 37.4(31.15, 42.3) | 35.23(33.1, 40.67) | 32.76(32.03, 38.9) | 32.46(32.44, 38.67) | **-0.249** | **0.001** |
| 6-10 years | 42.83(41.3, 45.91) | 41.33(37.44, 45.67) | 41.44(38.11, 45.69) | 42.7(39.82, 46.57) | 43.07(41.79, 46.12) | 41.68(40.94, 44.49) | 41.28(41.23, 44.1) | -0.016 | 0.657 |
| 11-14 years | 46.31(45.46, 47.8) | 45.44(43.32, 47.64) | 44.42(41.38, 47.65) | 45.43(42.79, 48.52) | 44.77(44.01, 47.45) | 43.9(43.18, 46.4) | 43.53(43.61, 46.08) | **-0.082** | **0.01** |
| 15-19 years | 46.39(44.26, 48.4) | 45.46(42.24, 48.06) | 45.02(42.3, 47.57) | 43.39(42.85, 47.06) | 44.43(44.56, 46.48) | 43.69(43.79, 45.74) | 44.1(44.29, 45.67) | **-0.084** | **0.023** |
| 20-24 years | 45.4(42.98, 48.9) | 43.17(39.01, 48.29) | 43.56(41.35, 47.53) | 43.3(42.48, 47.16) | 43.44(43.32, 45.71) | 43(43.01, 44.93) | 43.48(43.57, 45.27) | -0.048 | 0.142 |
| 25-29 years | 45.09(42.33, 48.57) | 43.34(37.68, 48.94) | 41.7(39.4, 47.81) | 42.09(41.19, 47.72) | 42.5(42.49, 46.49) | 41.99(41.84, 45.5) | 42.55(42.55, 45.68) | -0.073 | 0.109 |
| 30-34 years | 45.01(41.74, 48.24) | 43.99(38.13, 49.08) | 42.21(38.42, 48.42) | 42.05(39.64, 48.31) | 42.64(41.45, 47.12) | 42.15(41.32, 46.31) | 43.17(41.79, 46.22) | -0.069 | 0.123 |
| 35-39 years | 45.62(41.34, 48.94) | 44.38(38.35, 49.05) | 42.83(38.53, 48.67) | 42.61(39.12, 49.03) | 43.5(40.38, 47.96) | 42.79(40.81, 47.15) | 43.53(41.51, 47.08) | -0.068 | 0.11 |
| 40-44 years | 45.69(41.48, 49.05) | 44.61(37.72, 49.55) | 43.47(38.98, 48.76) | 43.36(39.51, 49.27) | 44.17(40.14, 49.02) | 43.81(40.37, 47.84) | 44.38(41.24, 47.83) | -0.038 | 0.259 |
| 45-49 years | 46.71(42.44, 50.11) | 44.52(38.81, 49.12) | 43.38(38.91, 49.19) | 43.92(40.16, 49.6) | 44.38(40.83, 49.33) | 44.51(40.52, 48.79) | 45.47(41.24, 48.7) | -0.024 | 0.628 |
| 50-54 years | 45.09(42.16, 49.26) | 45.5(40.07, 49.92) | 43.35(39.74, 48.79) | 43.89(40.4, 49.91) | 44.67(41.78, 49.46) | 44.68(41.5, 49.11) | 46.05(41.91, 49.65) | 0.016 | 0.702 |
| 55-59 years | 45.36(43.15, 49.52) | 44.2(40.36, 48.91) | 44.14(40.47, 49.28) | 43.99(41.38, 49.69) | 45.36(42.47, 50.18) | 44.97(42.51, 49.55) | 46.11(43.1, 49.67) | 0.035 | 0.302 |
| 60-64 years | 45.43(43.38, 48.46) | 44.34(40.89, 48.9) | 43.81(41.49, 48.4) | 44.68(42.3, 50.17) | 45.33(43.42, 50.16) | 45.77(43.24, 49.67) | 46.76(44.09, 50.01) | 0.06 | 0.126 |
| 65-69 years | 46.72(44.64, 48.82) | 45.03(42.07, 48.05) | 44.34(42.31, 48.02) | 44.04(43.44, 49.34) | 45.54(44.41, 50.36) | 45.8(44.4, 49.84) | 47.44(45.04, 50.35) | 0.032 | 0.555 |
| 70-74 years | 47.62(45.43, 49.99) | 45.99(42.88, 48.11) | 45.12(43.47, 47.76) | 44.65(44.26, 49.01) | 45.51(45.65, 49.86) | 45.83(45.38, 49.76) | 47.45(46.18, 50.41) | -0.008 | 0.878 |
| 75-79 years | 45.86(44.08, 47.65) | 45(43.88, 47.65) | 46.23(46.42, 47.65) | 47.41(47.31, 47.35) | 48.61(48.27, 48.33) | 48.55(48.12, 48.48) | 49.18(49.06, 49.1) | **0.145** | **0.002** |
| 80-84 years | 46.12(44.89, 47.76) | 45.7(45.05, 47.85) | 46.97(47.22, 48.55) | 48.54(47.18, 48.52) | 49.15(47.24, 49.2) | 49.67(48.66, 49.11) | 50.48(49.07, 49.89) | **0.173** | **<0.001** |
| 85-89 years | 45.68(43.71, 48.34) | 45.77(43.87, 48.8) | 47.66(45.88, 48.9) | 48.93(47.32, 49.44) | 48.55(46.79, 49.75) | 49.25(47.59, 50.01) | 50.45(48.72, 50.89) | **0.165** | **0.001** |
| 90-94 years | 44.42(42.63, 48.14) | 44.43(42.65, 48.32) | 46.43(44.7, 48.55) | 47.43(45.74, 48.63) | 48.42(46.72, 49.58) | 48.45(46.91, 49.57) | 49.12(47.61, 50.22) | **0.181** | **<0.001** |
| 95+ years | 42.8(40.84, 46.95) | 44.21(42.25, 48.33) | 45.9(44.28, 48.47) | 47.1(45.2, 48.37) | 47.64(45.96, 48.86) | 48.99(47.56, 50.05) | 49.26(47.98, 50.24) | **0.23** | **<0.001** |
| Rural | 46.19(42.26, 51.07) | 45.39(37.75, 52.45) | 44.3(37.89, 51.97) | 44.27(38.82, 52.74) | 43.66(40.11, 51.87) | 43.12(39.66, 50.61) | 43.33(40.3, 50.8) | **-0.104** | **0.001** |
| Urban | 42.13(40.56, 43.99) | 42.45(40.81, 44.59) | 41.95(42.03, 44.59) | 42.79(42.6, 44.95) | 43.8(42.92, 44.95) | 43.47(43.55, 45.14) | 44.12(44.28, 46.44) | **0.073** | **0.007** |
| Low (0-6 years formal) | 46.01(43.44, 48.72) | 47.22(41.41, 50.43) | 47.57(41.26, 52.39) | 47.63(41.1, 54.95) | 45.89(40.62, 53.49) | 43.5(38.64, 50.76) | 43.77(39.12, 50.99) | -0.116 | 0.08 |
| Medium (6.01-12 years) | 43.78(40.29, 46.08) | 41.73(37.84, 45.32) | 39.82(38.52, 44.56) | 40.06(38.95, 47.47) | 41.71(39.78, 48.07) | 42.85(42.29, 46.53) | 43.78(43.18, 47.2) | 0.026 | 0.717 |
| High (12.01+ years) | 40.8(37.63, 44.19) | 41.88(39.09, 45.78) | 43.67(41.64, 46.67) | 44.72(42.22, 46.91) | 44.83(42.55, 46.7) | 44.04(41.25, 47.98) | 44.61(41.9, 48.78) | **0.128** | **0.016** |
| East & Southeast Asia | 54.2(33.84, 56.38) | 47.34(30.74, 51.61) | 48.15(32.55, 50.81) | 48.38(34.15, 52.22) | 50.59(35.61, 53.68) | 49.4(35.35, 53.5) | 49.47(36.09, 53.32) | -0.059 | 0.561 |
| Central/Eastern Europe and central Asia | 34.91(29.66, 39.52) | 36.14(30.22, 41.35) | 36.83(29.37, 40.45) | 36.12(30.29, 42.24) | 35.95(31.33, 42.65) | 37.49(32.65, 43.69) | 37.36(32.97, 43.59) | **0.068** | **0.036** |
| High-Income Countries | 36.79(32.14, 42.38) | 37.63(31.14, 43.42) | 38.31(32.5, 44.28) | 38.57(32.42, 44.71) | 38.53(32.37, 44.78) | 38.26(33.02, 44.86) | 37.73(33.28, 46.28) | 0.034 | 0.196 |
| Latin America & Caribbean | 41.08(39.25, 40.61) | 43.82(42.81, 43.1) | 44.13(41.99, 43.21) | 43.5(42.01, 42.61) | 40.95(39.22, 42.07) | 41.23(39.52, 41.73) | 43.67(42.35, 42.74) | -0.008 | 0.905 |
| Middle East & North Africa | 39.36(38.97, 46.95) | 41.54(39.09, 48.27) | 39.87(38.27, 46.02) | 42.04(38.3, 46.63) | 42.55(39.02, 45.6) | 42.63(39.5, 44.09) | 43.18(40.13, 43.88) | **0.122** | **0.011** |
| South Asia | 55.7(45.12, 53.68) | 56.89(45.88, 55.54) | 58.13(46.5, 53.12) | 58.22(46.94, 57.3) | 59.53(48.03, 56.87) | 55.95(44.87, 50.99) | 56.84(45.9, 50.55) | 0.024 | 0.693 |
| Sub-Saharan Africa | 43.99(34.32, 50.71) | 44.76(37.17, 53.57) | 47.82(37.39, 54.89) | 46.71(39.2, 56.77) | 43.17(41.7, 58.48) | 39.84(39.52, 56.09) | 42.47(38.91, 53.54) | -0.143 | 0.196 |
| **Female** |  |  |  |  |  |  |  |  |  |
| Overall | 45.17(42.61, 47.56) | 44.35(40.65, 47.72) | 43.94(42.16, 47.46) | 43.67(43.24, 48.54) | 44.47(44.36, 48.13) | 44.06(43.91, 47.55) | 44.65(44.53, 47.82) | -0.014 | 0.542 |
| 0-11 mo. | 45.03(41.78, 42.83) | 45.45(38.59, 45.27) | 44.94(36.52, 46.2) | 44.67(34.67, 46.05) | 38.81(29.99, 45.02) | 37.84(29.05, 43.57) | 36.81(28.85, 42.73) | **-0.342** | **0.005** |
| 12-23 mo. | 39.81(37.31, 41.05) | 38.92(35.49, 42.55) | 38.24(34.32, 42.01) | 38.04(34.02, 40.73) | 35.93(31.09, 38.54) | 33.8(29.84, 36.79) | 33.66(30.38, 36.81) | **-0.231** | **<0.001** |
| 2-5 years | 40.56(35.05, 44.1) | 39.47(33.56, 43.7) | 39.14(32.62, 43.67) | 39.07(32.88, 43.77) | 36.88(35.11, 42.29) | 34.54(34.03, 40.72) | 34.46(34.39, 40.44) | **-0.226** | **0.001** |
| 6-10 years | 44.28(43.18, 47.48) | 42.65(39.26, 47.15) | 43.53(40.19, 47.32) | 44.77(41.75, 48.15) | 44.02(43.15, 47.16) | 42.7(42.22, 46.19) | 42.48(42.6, 45.9) | -0.034 | 0.384 |
| 11-14 years | 48.27(46.52, 49.48) | 47.21(44.18, 49.31) | 45.71(42.68, 49.34) | 46.45(44.07, 49.09) | 45.64(45.34, 48.23) | 44.76(44.34, 47.21) | 44.54(44.78, 47.04) | **-0.121** | **0.002** |
| 15-19 years | 47.36(45.3, 49.7) | 46.41(42.82, 48.65) | 45.94(43.6, 48.6) | 44.44(44.34, 47.86) | 45.71(45.89, 47.56) | 44.87(45.03, 46.77) | 45.24(45.46, 46.58) | **-0.074** | **0.04** |
| 20-24 years | 46.32(43.79, 50.46) | 44.15(40.35, 49.14) | 44.57(42.6, 48.35) | 44.32(43.88, 48.12) | 44.82(44.77, 46.59) | 44.26(44.3, 45.86) | 44.66(44.76, 45.94) | -0.035 | 0.268 |
| 25-29 years | 46.09(43.2, 50.38) | 43.98(38.88, 49.81) | 42.67(40.86, 48.92) | 43.16(42.57, 48.79) | 43.81(43.81, 47.28) | 43.33(43.21, 46.64) | 43.85(43.79, 46.65) | -0.053 | 0.246 |
| 30-34 years | 46.42(42.79, 50.13) | 44.93(38.93, 50.16) | 43.12(39.8, 49.62) | 43.29(41.06, 49.44) | 43.87(42.89, 48.06) | 43.52(42.59, 47.46) | 44.47(43.16, 47.47) | -0.062 | 0.196 |
| 35-39 years | 47.26(42.28, 50.88) | 45.54(39.35, 50.4) | 44.1(39.97, 50.05) | 43.8(40.55, 50.25) | 44.82(41.87, 49.22) | 44.22(42.26, 48.34) | 44.97(42.99, 48.29) | -0.068 | 0.152 |
| 40-44 years | 47.11(42.72, 51.08) | 45.76(38.65, 51.01) | 44.85(40.51, 50.42) | 44.52(41.06, 50.73) | 45.63(41.75, 50.28) | 45.22(41.87, 49.13) | 45.9(42.77, 49.22) | -0.032 | 0.39 |
| 45-49 years | 47.75(43.49, 51.36) | 45.99(40.41, 50.63) | 44.83(40.52, 50.71) | 45.24(41.89, 51.11) | 45.91(42.55, 50.64) | 46(42.14, 50.2) | 46.99(42.85, 50.32) | -0.012 | 0.791 |
| 50-54 years | 46(43.34, 50.26) | 46.68(41.58, 51.08) | 44.97(41.57, 50.43) | 45.26(42.29, 51.57) | 46.31(43.68, 51.09) | 46.26(43.3, 50.61) | 47.58(43.75, 50.86) | 0.036 | 0.335 |
| 55-59 years | 46.34(44.33, 50.3) | 45.47(42.51, 50.19) | 45.69(42.52, 50.75) | 45.69(43.6, 51.45) | 47.06(44.52, 51.79) | 46.67(44.56, 50.86) | 47.75(45.01, 51.22) | 0.058 | 0.07 |
| 60-64 years | 46.52(44.44, 48.9) | 46.13(43.05, 50.11) | 45.92(44.01, 49.98) | 46.18(44.61, 51.66) | 47.23(45.72, 51.93) | 47.35(45.49, 51.36) | 48.37(46.3, 51.74) | **0.067** | **0.033** |
| 65-69 years | 47.31(45.27, 49) | 46.86(44.18, 49.01) | 46.39(44.9, 49.62) | 46.15(46.35, 50.78) | 47.25(46.94, 51.79) | 47.59(46.75, 51.48) | 49.03(47.28, 51.84) | 0.053 | 0.178 |
| 70-74 years | 48.15(45.93, 49.87) | 47.57(44.87, 49.53) | 47.15(46.45, 49.63) | 46.89(47.28, 50.69) | 48.05(48.65, 50.98) | 47.65(47.94, 51.31) | 49.21(48.66, 52.08) | 0.029 | 0.39 |
| 75-79 years | 46.72(44.94, 48.73) | 46.75(46.23, 49.27) | 49.27(47.12, 49.51) | 49.8(47.1, 50.08) | 50.42(47.66, 51.04) | 50.7(48.04, 50.46) | 51.23(49.15, 51.02) | **0.169** | **0.001** |
| 80-84 years | 46.46(43.74, 49.34) | 46.72(44.09, 49.83) | 49.38(47.15, 50.24) | 48.91(46.08, 50.63) | 49.07(45.69, 50.97) | 50.37(47.85, 51.63) | 50.72(47.91, 52.28) | **0.148** | **0.003** |
| 85-89 years | 45.47(42.67, 49.17) | 45.46(42.67, 49.29) | 47.3(44.23, 49.48) | 48.44(45.76, 50.35) | 47.99(44.74, 50.01) | 48.98(45.82, 51.03) | 49.8(46.98, 51.97) | **0.154** | **0.001** |
| 90-94 years | 44.55(41.79, 48.33) | 44.41(41.43, 48.6) | 45.68(42.62, 48.54) | 46.04(43.09, 48.2) | 47.72(44.7, 49.68) | 47.96(45.05, 50.14) | 48.19(45.13, 50.48) | **0.15** | **<0.001** |
| 95+ years | 43.52(40.71, 48.44) | 44.52(41.3, 49.33) | 45.31(42.54, 48.9) | 45.56(42.85, 47.8) | 46.42(43.53, 48.51) | 48.57(45.93, 50.69) | 48.32(45.69, 50.49) | **0.176** | **<0.001** |
| Rural | 47.06(43.12, 51.65) | 45.49(38.65, 52.27) | 44.53(39.68, 51.99) | 44.58(40.66, 52.24) | 43.92(41.83, 51.86) | 43.38(41.26, 51.18) | 43.61(41.88, 51.33) | **-0.115** | **0.002** |
| Urban | 43.86(40.61, 45.86) | 44.41(40.93, 46.6) | 44.13(42.23, 46.71) | 45.15(42.9, 47.28) | 45.82(43.26, 47.3) | 45.7(44.74, 47.4) | 46.36(45.4, 48.67) | **0.087** | **0.001** |
| Low (0-6 years formal) | 47.47(44.23, 49.03) | 47.49(41.73, 51.08) | 47.52(41.47, 53.03) | 47.48(41.27, 53.66) | 45.72(40.57, 52.48) | 43.73(39.85, 51.2) | 44.06(40.39, 51.42) | **-0.145** | **0.009** |
| Medium (6.01-12 years) | 45.09(41.49, 47.44) | 43.24(39.84, 46.82) | 41.6(40.62, 46.29) | 41.96(41.04, 48.79) | 43.25(41.82, 49.39) | 44.85(44.44, 48.17) | 45.69(45.3, 48.82) | 0.046 | 0.517 |
| High (12.01+ years) | 41.8(37.84, 46.75) | 42.85(39.33, 48.41) | 44.95(41.69, 49.03) | 45.44(42.42, 49.34) | 45.47(42.71, 48.46) | 45.25(41.95, 49.59) | 45.80(42.6, 50.36) | **0.131** | **0.01** |
| East & Southeast Asia | 50.09(35.47, 57.65) | 44.12(33.64, 53) | 45.26(35.64, 52.89) | 46.44(37.48, 54.48) | 48.38(37.88, 55.94) | 47.99(37.44, 54.37) | 49.24(38.16, 53.69) | 0.059 | 0.542 |
| Central/Eastern Europe and central Asia | 37.13(29.62, 42.96) | 38.47(30.35, 47.72) | 38.76(30.19, 45.28) | 38.51(32.65, 45.26) | 38.26(33.64, 45.42) | 40.08(35.17, 46.63) | 39.8(35.37, 46.34) | **0.08** | **0.019** |
| High-Income Countries | 38.58(33.25, 43.32) | 39.54(32.26, 44.73) | 40.42(33.81, 46.19) | 40.64(33.42, 46.81) | 40.54(33.52, 47.68) | 40.24(34.16, 48.2) | 41.05(34.19, 49.52) | **0.067** | **0.02** |
| Latin America & Caribbean | 42.72(41.34, 41.92) | 45.6(43.77, 44.39) | 45.56(42.73, 45.18) | 44.48(42.01, 44.59) | 40.86(39.13, 42.59) | 41.1(39.38, 41.82) | 43.65(42.72, 43.58) | -0.085 | 0.308 |
| Middle East & North Africa | 42.18(39.27, 49.93) | 44.55(39.94, 51.12) | 42.35(39.43, 48.95) | 44.07(39.51, 49.49) | 44.38(39.98, 47.91) | 44.36(40.12, 46.79) | 44.73(40.6, 46.88) | 0.069 | 0.099 |
| South Asia | 57.59(47.03, 54.2) | 58.34(47.99, 55.25) | 60.81(49.02, 52.98) | 61.52(49.67, 58.01) | 63.17(51.21, 56.95) | 59.55(47.94, 52.88) | 60.59(49.13, 53.67) | 0.106 | 0.176 |
| Sub-Saharan Africa | 43.31(35.07, 51.77) | 43.99(37.92, 54.71) | 46.81(38.2, 56.1) | 45.37(40.08, 58.26) | 40.94(42.61, 57.78) | 38.77(40.4, 56.76) | 41.67(39.65, 55.02) | -0.161 | 0.148 |

**Supplementary Table 5. Absolute differences in PHDI scores between the highest and lowest subgroups of each demographic characteristic**

| **Year** | **PHDI** | **Whole grains** | **Whole fruit** | **Nonstarchy veg** | **Nuts seeds** | **Legumes** | **Unsaturated fat** | **Fish** | **Starchy veg** | **Dairy** | **Red meat** | **Egg** | **Saturated fat** | **Add sugars** |
| --- | --- | --- | --- | --- | --- | --- | --- | --- | --- | --- | --- | --- | --- | --- |
| **Female - Male** | | | | | | | | | | | | | | |
| 1990 | 1.191819 | 0.058386 | 0.957314 | 0.502176 | -0.00708 | -0.03005 | -0.19963 | -0.1859 | 0 | 0.706115 | 0 | -0.6095 | 0 | 0 |
| 1995 | 1.454631 | 0.043994 | 0.962091 | 0.510737 | -0.0045 | -0.03065 | -0.17972 | -0.18357 | 0 | 0.738784 | 0 | -0.40253 | 0 | 0 |
| 2000 | 1.571024 | 0.030988 | 0.99266 | 0.579408 | -0.0052 | -0.0097 | -0.17316 | -0.22827 | 0 | 0.734307 | 0 | -0.35001 | 0 | 0 |
| 2005 | 1.643858 | 0.012067 | 1.046545 | 0.632669 | 0.00524 | 0.017949 | -0.16836 | -0.2775 | 0 | 0.776247 | 0 | -0.40099 | 0 | 0 |
| 2010 | 1.988204 | -0.00428 | 1.00596 | 0.656456 | -0.01213 | 0.040272 | -0.13378 | -0.31314 | 0 | 0.748848 | 0 | 0 | 0 | 0 |
| 2015 | 1.921677 | -0.02197 | 0.966037 | 0.688449 | -0.02127 | 0.058864 | -0.09683 | -0.30513 | 0 | 0.653526 | 0 | 0 | 0 | 0 |
| 2018 | 1.8471 | -0.03075 | 0.961621 | 0.676971 | -0.01169 | 0.064302 | -0.07043 | -0.30945 | -0.07648 | 0.643006 | 0 | 0 | 0 | 0 |
| **Male, 80-84 years - 12-23 mo.** | | | | | | | | | | | | | | |
| 1990 | 7.525055 | 0.954002 | 3.579625 | 4.615362 | 0.165907 | 2.266213 | 6.627512 | 4.849927 | -9.05239 | 2.48754 | 0 | -8.96864 | 0 | 0 |
| 1995 | 8.027619 | 0.694208 | 3.695396 | 4.614763 | 0.098322 | 2.330712 | 6.872819 | 4.348892 | -8.65447 | 1.824903 | 0 | -7.79792 | 0 | 0 |
| 2000 | 10.08942 | 0.534628 | 3.917946 | 5.326456 | 0.185369 | 2.464589 | 7.358281 | 4.072337 | -8.00296 | 1.003936 | 0 | -6.77116 | 0 | 0 |
| 2005 | 11.9269 | 0.54817 | 4.39496 | 5.755928 | 0.162921 | 2.397698 | 7.127075 | 3.223256 | -7.3727 | 1.466451 | 0 | -5.77686 | 0 | 0 |
| 2010 | 14.61519 | 0.691525 | 4.177163 | 6.272051 | 0.00498 | 2.439687 | 7.028437 | 2.280894 | -5.50291 | 0.516106 | 0 | -3.29274 | 0 | 0 |
| 2015 | 17.46161 | 0.755631 | 4.138489 | 6.661643 | 0.056298 | 2.448466 | 7.301332 | 2.460694 | -4.77862 | 1.101316 | 0 | -2.68365 | 0 | 0 |
| 2018 | 18.42438 | 0.762092 | 4.343668 | 6.79282 | 0.26239 | 2.402587 | 7.312948 | 2.423929 | -4.9305 | 1.158791 | 0 | -2.10435 | 0 | 0 |
| **Female, 75-79 years - 12-23 mo.** | | | | | | | | | | | | | | |
| 1990 | 6.915644 | 1.142798 | 4.097623 | 4.918168 | 0.075158 | 2.007765 | 6.342011 | 4.613077 | -9.05201 | 2.284419 | 0 | -9.51336 | 0 | 0 |
| 1995 | 7.823019 | 0.769464 | 4.121082 | 5.01838 | 0.058798 | 2.223791 | 6.801587 | 4.084345 | -8.69584 | 1.287543 | 0 | -7.84613 | 0 | 0 |
| 2000 | 11.03196 | 0.810627 | 4.658957 | 6.043349 | 0.092915 | 2.1884 | 6.665376 | 3.626339 | -8.03563 | 1.555339 | 0 | -6.57371 | 0 | 0 |
| 2005 | 11.76558 | 0.828242 | 4.937067 | 6.599631 | 0.106952 | 2.233992 | 6.669077 | 2.713265 | -7.42368 | 0.692829 | 0 | -5.5918 | 0 | 0 |
| 2010 | 14.49038 | 0.759672 | 4.75741 | 6.748856 | -0.02761 | 2.408529 | 6.818491 | 1.759955 | -5.54014 | -0.13279 | 0 | -3.062 | 0 | 0 |
| 2015 | 16.90501 | 0.987251 | 4.585879 | 6.619868 | 0.020404 | 2.312501 | 6.878099 | 2.036017 | -5.02618 | 1.017134 | 0 | -2.52596 | 0 | 0 |
| 2018 | 17.57438 | 0.863676 | 4.726872 | 6.570195 | 0.243014 | 2.384364 | 7.031307 | 2.066804 | -5.19054 | 0.848527 | 0 | -1.96984 | 0 | 0 |
| **Male, Urban - Rural** | | | | | | | | | | | | | | |
| 1990 | -4.06004 | 0.000401 | 1.23011 | 0.470654 | 0.248997 | 0.050026 | -2.09318 | 0.049267 | -1.54224 | 4.239505 | 0 | -6.71358 | 0 | 0 |
| 1995 | -2.93844 | -0.06094 | 1.29768 | 0.187294 | 0.257087 | 0.014359 | -1.91085 | -0.85033 | -1.88165 | 4.261614 | 0 | -4.2527 | 0 | 0 |
| 2000 | -2.3536 | -0.11046 | 1.449503 | -0.16412 | 0.293851 | 0.026729 | -1.91385 | -0.71878 | -1.2797 | 4.142444 | 0 | -4.0792 | 0 | 0 |
| 2005 | -1.47566 | -0.24294 | 1.597262 | -0.24956 | 0.375417 | 0.020536 | -1.65617 | -0.74192 | -1.23864 | 3.841097 | 0 | -3.18074 | 0 | 0 |
| 2010 | 0.137565 | -0.38157 | 1.485679 | -0.334 | 0.419345 | 0.046608 | -1.47315 | -0.69678 | -0.94464 | 3.675742 | 0 | -1.65967 | 0 | 0 |
| 2015 | 0.351631 | -0.39669 | 1.538343 | -0.19123 | 0.50194 | 0.087653 | -1.33815 | -0.66762 | -0.76745 | 3.421948 | 0 | -1.83712 | 0 | 0 |
| 2018 | 0.794719 | -0.4187 | 1.618612 | -0.16658 | 0.589422 | 0.129809 | -1.32212 | -0.65384 | -1.03442 | 3.307986 | 0 | -1.25545 | 0 | 0 |
| **Female, Urban - Rural** | | | | | | | | | | | | | | |
| 1990 | -3.19858 | 0.110345 | 1.314199 | 0.591281 | 0.219808 | -0.09069 | -2.11426 | -0.70973 | -0.64445 | 4.418169 | 0 | -6.29324 | 0 | 0 |
| 1995 | -1.07562 | 0.043623 | 1.402405 | 0.293339 | 0.231509 | -0.11175 | -1.93618 | -0.64916 | -0.98258 | 4.455714 | 0 | -3.82253 | 0 | 0 |
| 2000 | -0.39884 | -0.00286 | 1.58654 | -0.06733 | 0.263836 | -0.08155 | -1.96198 | -0.51957 | -0.44881 | 4.343271 | 0 | -3.51037 | 0 | 0 |
| 2005 | 0.566689 | -0.13681 | 1.818894 | -0.13309 | 0.340349 | -0.05276 | -1.73572 | -0.52585 | -0.48855 | 4.064657 | 0 | -2.58442 | 0 | 0 |
| 2010 | 1.892772 | -0.27728 | 1.729377 | -0.21549 | 0.361066 | -0.0067 | -1.54898 | -0.44263 | -0.3636 | 3.600107 | 0 | -0.94312 | 0 | 0 |
| 2015 | 2.321044 | -0.30659 | 1.787098 | -0.08121 | 0.436265 | 0.040066 | -1.40596 | -0.40609 | -0.31829 | 3.627664 | 0 | -1.05191 | 0 | 0 |
| 2018 | 2.75615 | -0.33052 | 1.89526 | -0.06381 | 0.518184 | 0.082079 | -1.39009 | -0.39557 | -0.60846 | 3.517047 | 0 | -0.46798 | 0 | 0 |
| **Male, High (12.01+ years) - Low (0-6 years formal)** | | | | | | | | | | | | | | |
| 1990 | -4.06004 | 0.000401 | 1.23011 | 0.470654 | 0.248997 | 0.050026 | -2.09318 | 0.049267 | -1.54224 | 4.239505 | 0 | -6.71358 | 0 | 0 |
| 1995 | -2.93844 | -0.06094 | 1.29768 | 0.187294 | 0.257087 | 0.014359 | -1.91085 | -0.85033 | -1.88165 | 4.261614 | 0 | -4.2527 | 0 | 0 |
| 2000 | -2.3536 | -0.11046 | 1.449503 | -0.16412 | 0.293851 | 0.026729 | -1.91385 | -0.71878 | -1.2797 | 4.142444 | 0 | -4.0792 | 0 | 0 |
| 2005 | -1.47566 | -0.24294 | 1.597262 | -0.24956 | 0.375417 | 0.020536 | -1.65617 | -0.74192 | -1.23864 | 3.841097 | 0 | -3.18074 | 0 | 0 |
| 2010 | 0.137565 | -0.38157 | 1.485679 | -0.334 | 0.419345 | 0.046608 | -1.47315 | -0.69678 | -0.94464 | 3.675742 | 0 | -1.65967 | 0 | 0 |
| 2015 | 0.351631 | -0.39669 | 1.538343 | -0.19123 | 0.50194 | 0.087653 | -1.33815 | -0.66762 | -0.76745 | 3.421948 | 0 | -1.83712 | 0 | 0 |
| 2018 | 0.794719 | -0.4187 | 1.618612 | -0.16658 | 0.589422 | 0.129809 | -1.32212 | -0.65384 | -1.03442 | 3.307986 | 0 | -1.25545 | 0 | 0 |
| **Female, High (12.01+ years) - Low (0-6 years formal)** | | | | | | | | | | | | | | |
| 1990 | -3.19858 | 0.110345 | 1.314199 | 0.591281 | 0.219808 | -0.09069 | -2.11426 | -0.70973 | -0.64445 | 4.418169 | 0 | -6.29324 | 0 | 0 |
| 1995 | -1.07562 | 0.043623 | 1.402405 | 0.293339 | 0.231509 | -0.11175 | -1.93618 | -0.64916 | -0.98258 | 4.455714 | 0 | -3.82253 | 0 | 0 |
| 2000 | -0.39884 | -0.00286 | 1.58654 | -0.06733 | 0.263836 | -0.08155 | -1.96198 | -0.51957 | -0.44881 | 4.343271 | 0 | -3.51037 | 0 | 0 |
| 2005 | 0.566689 | -0.13681 | 1.818894 | -0.13309 | 0.340349 | -0.05276 | -1.73572 | -0.52585 | -0.48855 | 4.064657 | 0 | -2.58442 | 0 | 0 |
| 2010 | 1.892772 | -0.27728 | 1.729377 | -0.21549 | 0.361066 | -0.0067 | -1.54898 | -0.44263 | -0.3636 | 3.600107 | 0 | -0.94312 | 0 | 0 |
| 2015 | 2.321044 | -0.30659 | 1.787098 | -0.08121 | 0.436265 | 0.040066 | -1.40596 | -0.40609 | -0.31829 | 3.627664 | 0 | -1.05191 | 0 | 0 |
| 2018 | 2.75615 | -0.33052 | 1.89526 | -0.06381 | 0.518184 | 0.082079 | -1.39009 | -0.39557 | -0.60846 | 3.517047 | 0 | -0.46798 | 0 | 0 |
| **Male, South Asia - Central/Eastern Europe and central Asia** | | | | | | | | | | | | | | |
| 1990 | 20.78956 | 2.331774 | -2.75561 | 0.893005 | 0.086814 | 1.198785 | 9.034273 | -5.66035 | 7.934569 | -4.1911 | 9.215534 | 2.70187 | 0 | 0 |
| 1995 | 20.74452 | 2.399377 | -2.59209 | 1.081957 | -0.09288 | 1.489459 | 7.193527 | -5.28485 | 7.570822 | -3.27475 | 9.275312 | 2.978643 | 0 | 0 |
| 2000 | 21.29979 | 2.806033 | -2.47854 | 0.794865 | -0.17544 | 1.614756 | 7.038507 | -5.36675 | 6.673184 | -2.58941 | 9.580358 | 3.402216 | 0 | 0 |
| 2005 | 22.10428 | 3.035887 | -3.04088 | 0.209349 | -0.89345 | 1.655762 | 7.342816 | -4.47004 | 6.164191 | -0.80986 | 9.128638 | 3.781878 | 0 | 0 |
| 2010 | 23.5794 | 2.848522 | -3.01097 | -0.07962 | -1.01663 | 1.355101 | 8.078744 | -3.41169 | 5.024286 | 0.228308 | 9.223503 | 4.339849 | 0 | 0 |
| 2015 | 18.45817 | 2.944341 | -3.7537 | -1.13436 | -1.42624 | 1.1432 | 8.476746 | -4.04406 | 4.911313 | -1.07023 | 8.113105 | 4.29805 | 0 | 0 |
| 2018 | 19.48217 | 2.97892 | -4.03027 | -0.77745 | -2.30674 | 1.071702 | 8.502676 | -3.55698 | 4.892521 | -0.22984 | 8.263201 | 4.674437 | 0 | 0 |
| **Female, South Asia -Central/Eastern Europe and central Asia** | | | | | | | | | | | | | | |
| 1990 | 20.46426 | 1.866027 | -4.09743 | 0.894874 | 0.097113 | 1.427605 | 7.545937 | -4.34604 | 7.547338 | -3.27754 | 9.630453 | 3.175912 | 0 | 0 |
| 1995 | 19.86504 | 1.931561 | -3.90575 | 1.126397 | -0.1165 | 1.695295 | 6.005645 | -4.32519 | 7.209113 | -2.23355 | 8.980168 | 3.497847 | 0 | 0 |
| 2000 | 22.05352 | 2.331772 | -3.80117 | 0.702862 | -0.20579 | 1.831111 | 6.087956 | -3.7673 | 6.429366 | -1.48924 | 9.944637 | 3.989305 | 0 | 0 |
| 2005 | 23.00872 | 2.563558 | -4.53744 | -0.19573 | -0.96891 | 1.875729 | 6.437535 | -2.81384 | 5.944006 | 0.463272 | 9.821729 | 4.41882 | 0 | 0 |
| 2010 | 24.91093 | 2.412717 | -4.55474 | -0.3823 | -1.10469 | 1.581399 | 7.131333 | -1.64199 | 4.861886 | 1.627959 | 9.904529 | 5.074824 | 0 | 0 |
| 2015 | 19.47195 | 2.509499 | -5.27539 | -1.57434 | -1.50998 | 1.365346 | 7.524247 | -2.46339 | 4.797308 | 0.18071 | 8.929367 | 4.988564 | 0 | 0 |
| 2018 | 20.78954 | 2.543334 | -5.58294 | -1.03384 | -2.41884 | 1.295294 | 7.579713 | -1.96317 | 4.763816 | 1.087587 | 9.108578 | 5.409998 | 0 | 0 |

**Supplementary Table 6. Absolute differences in PHDI scores between 2018 and 1990 of each age groups.**

| **Age** | **PHDI** | **Whole grains** | **Whole fruit** | **Nonstarchy veg** | **Nuts seeds** | **Legumes** | **Unsaturated fat** | **Fish** | **Starchy veg** | **Dairy** | **Red meat** | **Egg** | **Saturated fat** | **Add sugars** |
| --- | --- | --- | --- | --- | --- | --- | --- | --- | --- | --- | --- | --- | --- | --- |
| **Male** |  |  |  |  |  |  |  |  |  |  |  |  |  |  |
| All ages | -1.17727 | 0.240379 | 0.38291 | 2.743349 | 1.383692 | 0.071967 | 0.041827 | -1.2108 | 0.076479 | 0.798406 | 0 | -5.70548 | 0 | 0 |
| 0-11 mo. | -7.97272 | 0.169407 | -0.15587 | 0.965356 | 1.385621 | -0.04983 | 0 | 0.502154 | -0.82239 | 1.928012 | -6.29281 | -5.60239 | 0 | 0 |
| 12-23 mo. | -6.53778 | 0.247014 | -0.06355 | 1.23404 | 1.360962 | -0.05082 | 0 | 0.955019 | -4.12189 | 1.764257 | 0 | -7.86281 | 0 | 0 |
| 2-5 years | -6.74446 | 0.337333 | 0.056408 | 1.538391 | 1.375383 | -0.03355 | -0.69078 | 1.629679 | -4.2015 | 1.685269 | 0 | -8.44109 | 0 | 0 |
| 6-10 years | -1.54114 | 0.408079 | 0.213899 | 1.761305 | 1.275543 | -0.02523 | -0.34074 | 2.348036 | -0.44735 | 1.40022 | 0 | -8.1349 | 0 | 0 |
| 11-14 years | -2.77818 | 0.522832 | 0.235528 | 1.988005 | 1.174704 | 0.011372 | -0.12549 | -0.05172 | 0 | 1.164895 | 0 | -7.6983 | 0 | 0 |
| 15-19 years | -2.28377 | 0.690948 | 0.258408 | 2.528666 | 1.243749 | 0.127536 | -0.14876 | -1.2253 | 0 | 1.230407 | 0 | -6.98943 | 0 | 0 |
| 20-24 years | -1.91657 | 0.701478 | 0.292056 | 2.819151 | 1.336419 | 0.149383 | -0.25761 | -1.2722 | 0 | 1.18254 | 0 | -6.86779 | 0 | 0 |
| 25-29 years | -2.54125 | 0.478387 | 0.342049 | 2.956179 | 1.415868 | 0.067484 | -0.18276 | -1.08842 | -0.72228 | 0.821887 | 0 | -6.62964 | 0 | 0 |
| 30-34 years | -1.8441 | 0.331856 | 0.313981 | 2.959344 | 1.434635 | 0.027084 | -0.06439 | -0.94684 | -0.10427 | 0.568409 | 0 | -6.36391 | 0 | 0 |
| 35-39 years | -2.08689 | 0.423124 | 0.298493 | 2.897411 | 1.421594 | 0.093327 | -0.17663 | -0.85385 | -0.37939 | 0.715405 | 0 | -6.52638 | 0 | 0 |
| 40-44 years | -1.31371 | 0.270062 | 0.237257 | 2.920049 | 1.396578 | 0.026502 | 0.046522 | -1.25043 | 0.905693 | 0.38229 | 0 | -6.24823 | 0 | 0 |
| 45-49 years | -1.24725 | 0.047299 | 0.286045 | 3.2269 | 1.377389 | -0.04448 | 0.055434 | -1.42466 | 1.616561 | 0.354589 | 0 | -6.74234 | 0 | 0 |
| 50-54 years | 0.966098 | -0.04666 | 0.295213 | 3.295087 | 1.407947 | 0.00756 | 0.276236 | -1.40914 | 2.866296 | 0.336069 | 0 | -6.06251 | 0 | 0 |
| 55-59 years | 0.752013 | 0.114684 | 0.346787 | 3.204209 | 1.40532 | 0.00282 | 0.098215 | -1.35334 | 2.542913 | 0.639991 | 0 | -6.24958 | 0 | 0 |
| 60-64 years | 1.321115 | 0.054927 | 0.352872 | 3.283211 | 1.394761 | 0.002553 | 0.27995 | -1.32166 | 2.504354 | 0.486691 | 0 | -5.71655 | 0 | 0 |
| 65-69 years | 0.715552 | 0.068279 | 0.388874 | 3.493221 | 1.383404 | -0.03057 | 0.244138 | -1.49611 | 2.291984 | 0.495889 | 0 | -6.12356 | 0 | 0 |
| 70-74 years | -0.167 | 0.149326 | 0.592576 | 3.445472 | 1.313536 | 0.007054 | -0.1007 | -1.55135 | 1.399556 | 1.185565 | 0 | -6.60804 | 0 | 0 |
| 75-79 years | 3.328308 | 0.138144 | 0.555284 | 3.633751 | 1.505886 | 0.094556 | 0.429081 | -1.55671 | 0 | 0.672013 | 0 | -2.1437 | 0 | 0 |
| 80-84 years | 4.361536 | 0.055104 | 0.700492 | 3.411498 | 1.457444 | 0.085552 | 0.685435 | -1.47098 | 0 | 0.435508 | 0 | -0.99852 | 0 | 0 |
| 85-89 years | 4.762257 | 0.028699 | 0.824207 | 3.190409 | 1.434 | 0.199095 | 0.935479 | -1.43745 | 0 | -0.15483 | 0 | -0.25736 | 0 | 0 |
| 90-94 years | 4.706914 | 0.068513 | 0.93911 | 2.741295 | 1.426463 | 0.228252 | 0.915993 | -1.26861 | 0 | -0.3441 | 0 | 0 | 0 | 0 |
| 95+ years | 6.456001 | 0.029489 | 1.113914 | 2.680285 | 1.514009 | 0.687625 | 1.527696 | -1.48093 | 0 | 0.383913 | 0 | 0 | 0 | 0 |
| **Female** |  |  |  |  |  |  |  |  |  |  |  |  |  |  |
| All ages | -0.52199 | 0.151241 | 0.387217 | 2.918145 | 1.379086 | 0.166323 | 0.17102 | -1.33434 | 0 | 0.735296 | 0 | -5.09598 | 0 | 0 |
| 0-11 mo. | -8.22112 | 0.186562 | -0.2838 | 0.996626 | 1.394232 | -0.05558 | 0 | 0.518207 | -0.4467 | 1.419201 | -5.94819 | -6.00168 | 0 | 0 |
| 12-23 mo. | -6.15085 | 0.244994 | -0.18532 | 1.276871 | 1.374022 | -0.05812 | 0 | 0.986653 | -3.86148 | 1.830922 | 0 | -7.75939 | 0 | 0 |
| 2-5 years | -6.09516 | 0.33222 | -0.0592 | 1.592816 | 1.381122 | -0.03986 | -0.59166 | 1.709446 | -3.82457 | 1.751711 | 0 | -8.34718 | 0 | 0 |
| 6-10 years | -1.79378 | 0.433312 | 0.109523 | 1.81812 | 1.279252 | -0.01742 | -0.26538 | 1.353949 | -0.06026 | 1.447112 | 0 | -7.89199 | 0 | 0 |
| 11-14 years | -3.73432 | 0.535563 | 0.125054 | 2.045589 | 1.182703 | 0.032195 | -0.09426 | -1.18085 | 0 | 1.214088 | 0 | -7.5944 | 0 | 0 |
| 15-19 years | -2.11711 | 0.680785 | 0.113698 | 2.625217 | 1.263882 | 0.129698 | -0.10645 | -1.37054 | 0 | 1.279462 | 0 | -6.73286 | 0 | 0 |
| 20-24 years | -1.66036 | 0.682349 | 0.157791 | 2.978118 | 1.342679 | 0.14984 | -0.15467 | -1.41291 | 0 | 1.240273 | 0 | -6.64383 | 0 | 0 |
| 25-29 years | -2.24219 | 0.469604 | 0.261821 | 3.158056 | 1.405132 | 0.068705 | -0.06533 | -1.37427 | -0.54989 | 0.830194 | 0 | -6.44621 | 0 | 0 |
| 30-34 years | -1.94393 | 0.32754 | 0.251854 | 3.19769 | 1.404692 | 0.030841 | 0.070716 | -1.40272 | -0.14588 | 0.540837 | 0 | -6.21949 | 0 | 0 |
| 35-39 years | -2.28487 | 0.418087 | 0.194173 | 3.120018 | 1.388465 | 0.100425 | -0.06316 | -1.39261 | -0.39081 | 0.698997 | 0 | -6.35845 | 0 | 0 |
| 40-44 years | -1.2092 | 0.254066 | 0.182787 | 3.092688 | 1.365449 | 0.032992 | 0.145915 | -1.31966 | 0.732176 | 0.360822 | 0 | -6.05643 | 0 | 0 |
| 45-49 years | -0.75937 | 0.020338 | 0.294836 | 3.346868 | 1.352833 | -0.04415 | 0.194353 | -1.43686 | 1.586184 | 0.28645 | 0 | -6.36023 | 0 | 0 |
| 50-54 years | 1.578755 | -0.12472 | 0.294612 | 3.357645 | 1.384183 | 0.013855 | 0.397148 | -1.40834 | 2.992809 | 0.205097 | 0 | -5.53353 | 0 | 0 |
| 55-59 years | 1.409111 | 0.061554 | 0.347536 | 3.25472 | 1.38764 | -0.00171 | 0.216193 | -1.3509 | 2.63358 | 0.561383 | 0 | -5.70088 | 0 | 0 |
| 60-64 years | 1.854078 | -0.02187 | 0.344418 | 3.312461 | 1.403656 | 0.037759 | 0.476618 | -1.29456 | 2.101452 | 0.28353 | 0 | -4.78939 | 0 | 0 |
| 65-69 years | 1.718894 | -0.04943 | 0.346226 | 3.538694 | 1.411841 | 0.05616 | 0.513458 | -1.39114 | 1.696621 | 0.188103 | 0 | -4.59163 | 0 | 0 |
| 70-74 years | 1.060677 | 0.071646 | 0.511935 | 3.506735 | 1.329787 | 0.095564 | 0.185469 | -1.39999 | 0.848785 | 0.869097 | 0 | -4.95835 | 0 | 0 |
| 75-79 years | 4.507892 | -0.03413 | 0.443929 | 2.928898 | 1.541877 | 0.318479 | 0.689296 | -1.55962 | 0 | 0.395029 | 0 | -0.21587 | 0 | 0 |
| 80-84 years | 4.260262 | -0.10031 | 0.665751 | 2.855063 | 1.496035 | 0.403761 | 0.880977 | -1.69402 | 0 | -0.24699 | 0 | 0 | 0 | 0 |
| 85-89 years | 4.330146 | -0.29051 | 0.593987 | 2.81133 | 1.46737 | 0.592375 | 1.05533 | -1.82174 | 0 | -0.078 | 0 | 0 | 0 | 0 |
| 90-94 years | 3.643376 | -0.26625 | 0.360035 | 2.701891 | 1.372306 | 0.659186 | 1.042795 | -1.94085 | 0 | -0.28574 | 0 | 0 | 0 | 0 |
| 95+ years | 4.792248 | -0.5041 | 0.507341 | 2.765259 | 1.410737 | 1.154112 | 1.405952 | -2.34992 | 0 | 0.402861 | 0 | 0 | 0 | 0 |

**Supplementary Table 7. Planetary health diet index score and rankings for 185 countries in 2018.**

| **Rank** | **Country** | **PHDI score(95%UI)** | **Country** | **PHDI score(95%UI)** | **Country** | **PHDI score(95%UI)** |
| --- | --- | --- | --- | --- | --- | --- |
|  | **Total** | | **Male** | | **Female** | |
| 1 | Samoa | 74.35(60.3, 60.91) | Samoa | 72.92(63.22, 60.06) | Samoa | 73.98(61.13, 58.98) |
| 2 | Afghanistan | 68.4(46.48, 64.81) | Sri Lanka | 67.14(58.08, 55.23) | Afghanistan | 66.84(42.12, 65.65) |
| 3 | Sri Lanka | 67.33(56.82, 53.87) | Maldives | 66.69(71.61, 53.24) | Sri Lanka | 66.69(55.88, 53.98) |
| 4 | Maldives | 64.6(70.97, 53.05) | Afghanistan | 66.58(43.05, 65.86) | Solomon Islands | 65.26(53.99, 45.53) |
| 5 | Solomon Islands | 64.25(56.99, 47.3) | Bhutan | 64.77(64.77, 55.36) | Maldives | 62.64(67.43, 49.32) |
| 6 | Bhutan | 63.87(67.69, 56.62) | Solomon Islands | 64.66(51.51, 44.89) | Bhutan | 62.53(63.2, 54.01) |
| 7 | Iran | 60.82(62.8, 57.72) | Iran | 60.08(63.33, 57.02) | Iran | 61.54(65.9, 58.03) |
| 8 | Bangladesh | 58.84(56.07, 54.07) | Bangladesh | 59.67(56.02, 53.92) | Bangladesh | 58.2(55.43, 54.03) |
| 9 | Tanzania | 57.73(64.45, 53.88) | Tanzania | 59.41(64.13, 55.61) | Lebanon | 57.86(58.04, 53.51) |
| 10 | China | 57.18(53.33, 35.03) | China | 56.88(50.98, 34.64) | China | 57.78(52.84, 35.1) |
| 11 | Niger | 56.25(47.87, 65) | Guinea-Bissau | 56.54(48.11, 56.68) | Tanzania | 57.73(63.61, 52.81) |
| 12 | Guinea-Bissau | 56.24(51.3, 54.49) | Panama | 55.26(55.34, 50.46) | India | 57.08(50.42, 50.75) |
| 13 | Rwanda | 55.5(51.85, 61.58) | Saint Vincent and the Grenadines | 55.14(51.57, 35.74) | Niger | 56.48(45.56, 66.33) |
| 14 | Panama | 55.23(60.76, 49.94) | Niger | 54.38(44.91, 66.94) | Serbia | 56.47(64.84, 43.65) |
| 15 | India | 55.12(49.19, 50.52) | Rwanda | 54.05(51.36, 62.23) | Guinea-Bissau | 56.11(48.47, 56.4) |
| 16 | Serbia | 55.11(63.38, 43.03) | Serbia | 53.72(61.84, 41.51) | Panama | 56.03(56.61, 49.5) |
| 17 | Saint Vincent and the Grenadines | 54.98(56.43, 37.86) | Cape Verde | 53.63(62.99, 52.06) | Rwanda | 55.89(54.42, 63.69) |
| 18 | Cape Verde | 54.08(64.27, 52.75) | Jordan | 53.52(46.31, 44.54) | Saint Vincent and the Grenadines | 55.68(52.44, 34.41) |
| 19 | Lebanon | 53.46(55.23, 49.58) | India | 52.77(46.84, 46.81) | Cape Verde | 54.1(62.3, 50.74) |
| 20 | Jordan | 52.66(46.61, 44.81) | Central African Republic | 52.28(46.83, 45.49) | South Korea | 53.77(51.54, 53.8) |
| 21 | Malawi | 52.5(54.86, 39.61) | Japan | 51.91(57.2, 48.83) | Malawi | 52.52(57.88, 41.06) |
| 22 | South Korea | 52.27(49.46, 53.02) | Burkina Faso | 51.85(49.33, 42.12) | Japan | 52.3(60.56, 46.76) |
| 23 | Central African Republic | 52.22(48.89, 44.06) | Malawi | 51.73(57.8, 41.31) | Brazil | 52.11(56.05, 45.91) |
| 24 | Japan | 52.19(58.72, 50.12) | Taiwan (Chinese province) | 51.46(47.59, 36.92) | Taiwan (Chinese province) | 52.08(45.54, 37.29) |
| 25 | Taiwan (Chinese province) | 51.82(46.21, 38.11) | France | 50.23(60.13, 44.76) | Jordan | 51.94(45.1, 44.7) |
| 26 | Brazil | 50.97(54.58, 45.42) | Moldova | 49.82(56.46, 46.24) | Central African Republic | 51.89(46.95, 46.08) |
| 27 | Burkina Faso | 50.8(50.02, 42.9) | Palestine | 49.77(49.05, 42.55) | Palestine | 51.38(51.01, 43.32) |
| 28 | France | 50.69(57.74, 48.14) | Lebanon | 49.77(52.02, 48.73) | Bulgaria | 51.37(58.24, 40.43) |
| 29 | Palestine | 50.68(47.54, 45) | Brazil | 49.71(54.09, 44.7) | Burkina Faso | 50.51(48.76, 42.72) |
| 30 | Moldova | 50.21(55.34, 47.2) | Sierra Leone | 49.49(54.02, 33.7) | Fiji | 50.49(50.55, 41.43) |
| 31 | Saint Lucia | 50.1(46.78, 48.74) | Nepal | 49.2(57.13, 59.17) | Marshall Islands | 50.43(46.78, 39.27) |
| 32 | Bulgaria | 49.98(57.62, 40.12) | Uruguay | 49.16(43.66, 41.11) | United Arab Emirates | 50.38(53, 44.67) |
| 33 | Marshall Islands | 49.55(44.08, 38.72) | Saint Lucia | 49.06(41.4, 45.19) | Moldova | 50.21(59.59, 47.04) |
| 34 | Fiji | 49.51(49.39, 40.83) | Fiji | 48.88(48.08, 41.22) | Saint Lucia | 50.16(42.03, 43.88) |
| 35 | Nepal | 49.08(54.53, 58.71) | South Korea | 48.83(46.65, 51.76) | Kenya | 49.64(55, 42.67) |
| 36 | Belgium | 48.73(45.33, 40.81) | The Bahamas | 48.82(39.58, 38.48) | Uruguay | 49.61(45.49, 42.86) |
| 37 | Madagascar | 48.68(46.83, 44.23) | Madagascar | 48.75(46.6, 45.26) | Belgium | 49.56(47.09, 41.62) |
| 38 | United Arab Emirates | 48.64(49.07, 42.19) | Bulgaria | 48.51(56.64, 37.53) | Netherlands | 49.41(53.71, 50.34) |
| 39 | Kenya | 48.47(55.62, 42.96) | Angola | 48.18(55.21, 53.66) | Madagascar | 48.88(46.8, 44.04) |
| 40 | Uruguay | 48.41(49.87, 42.1) | Kenya | 48.1(56.46, 43.6) | Suriname | 48.6(54.71, 53.55) |
| 41 | Netherlands | 48.35(52.75, 49.08) | Grenada | 47.93(48.8, 45.44) | Mali | 48.56(50.81, 41.09) |
| 42 | Tonga | 48.29(44.49, 36.26) | Belgium | 47.91(44.74, 38.58) | Nepal | 48.49(55.09, 59.67) |
| 43 | Suriname | 48.1(55.92, 54.44) | Zambia | 47.87(62.84, 41.39) | France | 48.47(53.32, 47.27) |
| 44 | Mali | 47.81(51.84, 41.98) | Suriname | 47.79(53.34, 54.73) | Cyprus | 48.46(54.02, 39.38) |
| 45 | Zambia | 47.73(63.21, 40.36) | Botswana | 47.41(57.85, 38.99) | The Bahamas | 48.43(39.98, 39.28) |
| 46 | Angola | 47.64(54.17, 52.6) | United Arab Emirates | 47.35(47.91, 39.76) | Syria | 48.39(51.03, 38.63) |
| 47 | Syria | 47.58(47.26, 39.86) | Netherlands | 47.09(54.12, 47.29) | Grenada | 48.33(50.62, 44.06) |
| 48 | The Bahamas | 47.58(43.03, 40.06) | Mali | 46.98(51.13, 45.32) | Tonga | 48.29(49.44, 37.53) |
| 49 | Grenada | 47.34(49.51, 47.09) | Marshall Islands | 46.8(48.76, 38.46) | Turkmenistan | 48.04(56.38, 46.81) |
| 50 | Cyprus | 47.26(48.97, 39.85) | Uganda | 46.76(58.89, 43.23) | Zambia | 47.94(62.05, 40.92) |
| 51 | Saudi Arabia | 47.21(47.69, 40.22) | Syria | 46.65(49.26, 36.51) | Angola | 47.6(54.59, 54.31) |
| 52 | Nigeria | 47.15(52.75, 35.95) | Nigeria | 46.5(52.13, 39.74) | Saudi Arabia | 47.54(51.96, 39.18) |
| 53 | Botswana | 47.11(59.01, 39.47) | Trinidad and Tobago | 46.35(52.77, 35.24) | Trinidad and Tobago | 47.26(53.2, 34.36) |
| 54 | Nicaragua | 46.95(50.66, 46.8) | Nicaragua | 46.06(50.65, 46.45) | Botswana | 47.26(57.85, 37.81) |
| 55 | Turkmenistan | 46.81(52.31, 45.54) | Turkmenistan | 45.88(52.47, 44.57) | Nigeria | 47.11(52, 37.72) |
| 56 | Trinidad and Tobago | 46.71(54.67, 37.65) | Tonga | 45.79(51.14, 35.45) | Nicaragua | 46.75(52.1, 45.7) |
| 57 | Uganda | 46.35(59.2, 40.76) | Cyprus | 45.76(52.03, 37) | Sierra Leone | 46.63(54.49, 33.04) |
| 58 | Sierra Leone | 46.03(56.14, 31.68) | Equatorial Guinea | 45.6(45.89, 51.11) | Philippines | 46.62(52.41, 43.85) |
| 59 | Singapore | 45.95(35.85, 32.84) | Zimbabwe | 45.32(56.73, 43.9) | Qatar | 46.56(55.26, 38.72) |
| 60 | Equatorial Guinea | 45.81(47.78, 47.77) | Saudi Arabia | 45.26(49.83, 39.26) | Azerbaijan | 46.46(43.99, 45.91) |
| 61 | Djibouti | 45.81(43.37, 46.97) | Djibouti | 45.23(41.88, 46.63) | Canada | 46.35(50.73, 43.57) |
| 62 | Canada | 45.66(47.41, 43.24) | Jamaica | 45.15(51.47, 30.54) | Bahrain | 46.34(53.53, 38.52) |
| 63 | Philippines | 45.62(52.24, 44.01) | Italy | 44.81(56.33, 38.63) | Italy | 46.34(55.5, 40.11) |
| 64 | Azerbaijan | 45.54(41.65, 45.07) | Chad | 44.71(46.92, 36.43) | Oman | 46.24(53.25, 41.72) |
| 65 | Zimbabwe | 45.35(56.34, 42.95) | Philippines | 44.48(54.95, 44.33) | Singapore | 46.24(37.38, 32.11) |
| 66 | Bahrain | 45.28(53.13, 39.53) | Canada | 44.47(48.06, 42.16) | Malta | 46.21(55.44, 32.08) |
| 67 | Oman | 45.14(50.29, 41.89) | Burundi | 44.43(56.33, 47.35) | Uganda | 46.15(59.02, 40.81) |
| 68 | Italy | 45.07(55.01, 39.45) | Vietnam | 44.42(52.26, 51.07) | Equatorial Guinea | 46(45.95, 49.62) |
| 69 | Qatar | 45.05(53.89, 39.66) | Qatar | 44.3(52.8, 38.86) | Jamaica | 45.84(53.27, 29.82) |
| 70 | Vietnam | 44.75(48.34, 52.58) | Oman | 44.23(51.3, 41.05) | Djibouti | 45.81(41.3, 45.43) |
| 71 | Jamaica | 44.75(52.55, 31.82) | Azerbaijan | 44.2(39.01, 44.67) | Zimbabwe | 45.43(56.38, 42.57) |
| 72 | Chad | 44.67(48.11, 33.6) | Bahrain | 44.16(52.06, 38.77) | Thailand | 45.24(40.81, 38.5) |
| 73 | Malta | 44.52(55.64, 30.75) | Sao Tome and Principe | 43.83(48.38, 30.52) | Malaysia | 45.18(44.05, 45.33) |
| 74 | Burundi | 44.07(55.93, 44.37) | Pakistan | 43.52(36.82, 37.45) | Estonia | 45.18(48.62, 40.01) |
| 75 | Thailand | 43.95(40.6, 37.79) | Singapore | 43.45(38.05, 30.98) | Chad | 45.12(46.87, 35.17) |
| 76 | Myanmar | 43.69(37.53, 38.42) | Vanuatu | 43.45(48.61, 30.8) | Vietnam | 44.67(47.37, 55.55) |
| 77 | Antigua and Barbuda | 43.63(54.73, 37.04) | Antigua and Barbuda | 43.39(52.61, 35.03) | Burundi | 44.48(55.98, 46.79) |
| 78 | Estonia | 43.62(47.09, 38.82) | Venezuela | 43.32(50.39, 40.95) | Myanmar | 44.3(36.34, 40.19) |
| 79 | Kazakhstan | 43.6(52.89, 37.24) | Malta | 42.97(54.34, 31.26) | Kazakhstan | 44.17(54.33, 37.57) |
| 80 | Malaysia | 43.55(46.13, 42.34) | Belarus | 42.95(44.73, 41.42) | Morocco | 44.03(54.51, 41.32) |
| 81 | Sao Tome and Principe | 43.44(48.96, 28.54) | Kazakhstan | 42.77(50.87, 37.24) | Antigua and Barbuda | 44(53.45, 34.46) |
| 82 | Belarus | 43.29(46.67, 40.57) | Sudan | 42.61(65.14, 42.71) | Barbados | 43.86(48.22, 39.18) |
| 83 | Morocco | 43.17(49.87, 38.32) | Timor-Leste | 42.6(50.27, 36.86) | Sao Tome and Principe | 43.57(49.55, 30.08) |
| 84 | Venezuela | 43.03(52.66, 41.34) | Malaysia | 42.43(47.76, 41.11) | Slovakia | 43.49(40.58, 37.72) |
| 85 | Pakistan | 42.98(35.43, 38.63) | Estonia | 42.07(45.79, 37) | United States | 42.97(52.8, 40.74) |
| 86 | Barbados | 42.8(50.13, 40.63) | Barbados | 41.98(46.91, 39.53) | Pakistan | 42.91(34.67, 36.81) |
| 87 | Timor-Leste | 42.29(48.15, 38.73) | Thailand | 41.9(38.4, 35.04) | Albania | 42.85(41.75, 41.13) |
| 88 | Dominica | 42.12(51.35, 37.83) | Namibia | 41.88(64.13, 48.62) | Belarus | 42.78(46, 41.65) |
| 89 | Namibia | 42.09(61.48, 46.96) | Dominica | 41.18(55.46, 36.89) | Timor-Leste | 42.72(48.03, 39.21) |
| 90 | Slovakia | 41.97(40.87, 36.57) | Dominican Republic | 41.07(46.89, 43.91) | Venezuela | 42.65(50.89, 43.13) |
| 91 | Albania | 41.68(40.13, 40.44) | Morocco | 41.04(49.49, 37.55) | Dominica | 42.3(57.33, 36.54) |
| 92 | Vanuatu | 41.51(48.26, 31.45) | Montenegro | 40.97(44.82, 38.22) | Namibia | 42.01(62.11, 47.97) |
| 93 | United States | 41.51(50.08, 39.94) | Myanmar | 40.87(36.76, 37.93) | Montenegro | 41.92(46.65, 39.01) |
| 94 | Belize | 41.46(45.05, 36.65) | Haiti | 40.79(56.2, 40.82) | Iraq | 41.91(49.21, 38.74) |
| 95 | Montenegro | 41.44(43.63, 39.82) | United States | 40.76(50.56, 39.02) | Belize | 41.9(41.72, 34.66) |
| 96 | Sudan | 41.37(65.74, 41.46) | Ethiopia | 40.73(54.4, 32.94) | Vanuatu | 41.88(48.77, 32.94) |
| 97 | Federated States of Micronesia | 41.37(34.58, 37.6) | Cameroon | 40.58(55.82, 41.15) | Guinea | 41.79(37.1, 34.88) |
| 98 | Guinea | 41.15(37.49, 34.46) | Slovakia | 40.51(39.12, 34.73) | Macedonia | 41.61(50.75, 36.66) |
| 99 | Dominican Republic | 41.01(43.8, 42.16) | Belize | 40.42(40.94, 34.51) | Federated States of Micronesia | 41.5(35.17, 36.72) |
| 100 | Haiti | 40.65(52.02, 40.1) | Albania | 40.41(37.74, 40.94) | Sudan | 41.47(64.86, 42.55) |
| 101 | Colombia | 40.62(36.54, 41.48) | Colombia | 40.32(35.54, 41.93) | Denmark | 41.14(59.91, 26.11) |
| 102 | Mauritius | 40.25(52.69, 38.86) | Federated States of Micronesia | 40.19(32.45, 35.96) | Mauritius | 40.86(55.54, 39.98) |
| 103 | Macedonia | 40.18(49.89, 34.35) | Switzerland | 40.12(58.88, 31.65) | Colombia | 40.71(35.85, 42.9) |
| 104 | Iraq | 39.87(49.96, 40.31) | Guinea | 40(37.26, 37.62) | Haiti | 40.65(55.75, 40.98) |
| 105 | Switzerland | 39.84(58.67, 31.15) | The Gambia | 39.31(51.45, 35.25) | Dominican Republic | 40.64(44.84, 42.63) |
| 106 | Germany | 39.8(41.05, 37.72) | Macedonia | 39.31(48.01, 34.1) | Germany | 39.96(41.62, 38.02) |
| 107 | Cameroon | 39.38(55.62, 40.93) | Chile | 39.16(43.07, 47.5) | Gabon | 39.65(48.95, 44.84) |
| 108 | Democratic Republic of the Congo | 39.21(45.89, 46.54) | Romania | 39.16(48.76, 32.82) | Democratic Republic of the Congo | 39.61(46.75, 47.08) |
| 109 | Gabon | 39.2(50.92, 43.74) | Democratic Republic of the Congo | 39.14(48.1, 45.86) | Libya | 39.56(50.67, 31.49) |
| 110 | Chile | 39.08(42.15, 46.53) | Iraq | 39.13(46.93, 39.12) | Mongolia | 39.42(49.23, 35.23) |
| 111 | Libya | 39.07(48.66, 31.7) | Mexico | 39.13(39.15, 37.84) | Ecuador | 39.37(47.17, 34.47) |
| 112 | The Gambia | 38.87(51.17, 32.95) | Germany | 39.12(39.51, 37.51) | Algeria | 39.37(46.15, 34.39) |
| 113 | Ethiopia | 38.68(52.67, 32.81) | Costa Rica | 39.1(43.25, 34.84) | Hungary | 39.33(39.61, 22.85) |
| 114 | Mexico | 38.57(39.13, 37.28) | Gabon | 39.08(50.13, 44.19) | Cameroon | 39.26(55.66, 42.1) |
| 115 | Ecuador | 38.51(45.36, 34.48) | Mauritius | 38.99(55.8, 39.17) | Switzerland | 39.04(61.81, 31.41) |
| 116 | Romania | 38.49(48.36, 33.02) | Libya | 38.57(49.86, 30.6) | Ukraine | 38.97(44.23, 37.09) |
| 117 | Algeria | 38.33(39.44, 32.59) | Lesotho | 38.52(48.26, 41.87) | Ethiopia | 38.88(55.49, 33.06) |
| 118 | Costa Rica | 38.07(46.68, 34.84) | South Sudan | 38.01(57.41, 41.42) | Costa Rica | 38.86(43.87, 34.86) |
| 119 | Hungary | 38.05(37.37, 24.6) | Cote d'Ivoire | 37.7(53.79, 31.8) | New Zealand | 38.59(62.28, 35.98) |
| 120 | Denmark | 38(59.83, 25.9) | Mauritania | 37.63(55.31, 29.37) | Chile | 38.37(43.82, 48.37) |
| 121 | South Sudan | 38(57.27, 38.62) | Ecuador | 37.56(45.83, 33.88) | Kiribati | 38.22(29.92, 34.33) |
| 122 | Lesotho | 37.89(48.62, 41.82) | Denmark | 37.46(56.5, 22.77) | Greece | 38.11(58.65, 26.22) |
| 123 | Ukraine | 37.88(40.47, 34.54) | Mozambique | 37.45(49.58, 40.89) | Mauritania | 38.08(55.02, 27.87) |
| 124 | Mozambique | 37.85(47.9, 42.84) | Algeria | 37.31(42.7, 33.59) | Mexico | 38.07(38.96, 36.38) |
| 125 | Mongolia | 37.72(46.82, 34.29) | New Zealand | 37.25(58.88, 34.13) | Mozambique | 38.06(48.05, 44.13) |
| 126 | Kiribati | 37.56(30.21, 34.64) | Benin | 37.24(56.95, 42.78) | Australia | 38.04(67.84, 33.27) |
| 127 | El Salvador | 37.53(44.05, 43.88) | Hungary | 37.13(36.89, 23.2) | Brunei | 38.01(32.85, 34.44) |
| 128 | Australia | 37.09(60.1, 33.01) | Cambodia | 37.03(38.95, 31.48) | Finland | 37.99(57.77, 27.91) |
| 129 | Mauritania | 37.08(56.52, 30.1) | Australia | 37.01(65.71, 32.84) | Czech Republic | 37.98(44.5, 22.61) |
| 130 | New Zealand | 37.08(56.06, 36.22) | Mongolia | 37.01(44.55, 33.61) | Romania | 37.96(50, 32.59) |
| 131 | Brunei | 36.89(33.11, 32.26) | Togo | 36.8(41.77, 32.02) | The Gambia | 37.88(52.36, 34.23) |
| 132 | Greece | 36.79(54.24, 24.93) | Ukraine | 36.69(38.03, 34.31) | El Salvador | 37.75(46.57, 44.32) |
| 133 | Togo | 36.65(41.42, 30.52) | Greece | 36.52(55.66, 23.73) | Lesotho | 37.73(50.32, 43.94) |
| 134 | Czech Republic | 36.65(40.5, 22.22) | El Salvador | 36.15(45.82, 44.56) | South Sudan | 37.71(57.13, 42.46) |
| 135 | Benin | 36.61(54.26, 41.32) | Poland | 36.07(35.37, 34.63) | Cambodia | 37.65(31.99, 32.46) |
| 136 | Latvia | 36.3(45.93, 28.54) | Czech Republic | 36.04(40.13, 20.26) | Latvia | 37.33(46.76, 28.92) |
| 137 | Argentina | 36.22(39.3, 31.5) | Latvia | 35.71(43.71, 27.73) | Laos | 37(44.7, 33.94) |
| 138 | Cambodia | 36.17(33.43, 33.29) | Guatemala | 35.71(37.28, 38.8) | Argentina | 36.92(39.93, 31.83) |
| 139 | Finland | 36.15(53.18, 27.8) | Kiribati | 35.58(28.49, 33.79) | Uzbekistan | 36.83(48.2, 31.86) |
| 140 | Cote d'Ivoire | 36.07(51.93, 29.98) | Guyana | 35.53(49.07, 36.83) | Russia | 36.72(48.54, 33.94) |
| 141 | Guyana | 36.03(44.89, 36.1) | Argentina | 35.43(38.46, 30.57) | Benin | 36.64(56.76, 43.79) |
| 142 | Seychelles | 35.81(52.3, 41.5) | Seychelles | 35.41(49.31, 42.99) | Austria | 36.43(47.94, 29.92) |
| 143 | Uzbekistan | 35.71(46.13, 31.43) | Cuba | 35.34(34.38, 35.72) | Togo | 36.41(41.78, 32.88) |
| 144 | Bolivia | 35.6(36.04, 43.9) | Bolivia | 35.26(36.98, 45.82) | Guyana | 36.35(46.43, 36.22) |
| 145 | Austria | 35.54(42.68, 28.55) | Laos | 35.23(41.59, 31.66) | Seychelles | 36.17(52.27, 44.2) |
| 146 | Russia | 35.43(46, 32.56) | Brunei | 35.17(31.44, 30.58) | Poland | 35.62(38.77, 35.15) |
| 147 | Laos | 35.36(42.76, 34.06) | Uzbekistan | 35.02(45.1, 31.82) | Cote d'Ivoire | 35.47(52.24, 32.51) |
| 148 | Poland | 35.15(34.66, 35.51) | Austria | 34.99(42.06, 26.55) | Papua New Guinea | 35.44(34.2, 31.34) |
| 149 | Papua New Guinea | 34.96(35.38, 32.71) | Ghana | 34.96(57.24, 35.09) | Bolivia | 35.39(35.54, 45.63) |
| 150 | Ghana | 34.94(53.54, 35.94) | Papua New Guinea | 34.65(32.55, 30.97) | Turkey | 35.31(53.86, 26.9) |
| 151 | Guatemala | 34.93(37.64, 38.41) | Russia | 34.59(45.84, 31.03) | Tunisia | 35.2(40.23, 23.1) |
| 152 | Cuba | 34.2(38.1, 35.17) | Finland | 33.98(52.3, 26.54) | United Kingdom | 35.06(56.29, 31.3) |
| 153 | Turkey | 34.14(53.1, 25.74) | Indonesia | 33.82(42.9, 34.06) | Cuba | 35.05(35.74, 35.35) |
| 154 | Peru | 34.01(41.65, 37.25) | Portugal | 33.41(46.05, 27.24) | Ghana | 35.03(51.68, 36.9) |
| 155 | Paraguay | 33.94(35.33, 40.69) | Peru | 33.32(44.33, 37.85) | Israel | 34.86(42.6, 35.84) |
| 156 | Swaziland | 33.87(34.17, 40.33) | Swaziland | 33.25(33.62, 40.95) | Peru | 34.59(45.34, 39.49) |
| 157 | Tunisia | 33.85(36.82, 22.73) | Slovenia | 33.03(42.45, 27.07) | Guatemala | 34.48(36.72, 40.31) |
| 158 | Slovenia | 33.53(43.98, 27.64) | Turkey | 32.94(52.8, 24.82) | Slovenia | 34.47(45.4, 29.57) |
| 159 | United Kingdom | 33.32(49.39, 29.58) | Paraguay | 32.93(38.56, 40.26) | Swaziland | 34.31(34.35, 40.32) |
| 160 | Indonesia | 33.29(38.34, 33.82) | United Kingdom | 32.88(53.87, 27.9) | Paraguay | 34.21(38.79, 40.34) |
| 161 | Congo | 33.15(43.09, 42.08) | Tunisia | 32.66(36.15, 22) | Yemen | 33.78(46.65, 43.23) |
| 162 | Portugal | 33.1(43.55, 28.12) | Congo | 32.3(44.54, 44.16) | Congo | 33.54(43.84, 44.22) |
| 163 | Yemen | 32.85(42.42, 40.35) | Yemen | 31.59(46.78, 41.6) | Portugal | 32.84(45.26, 28.41) |
| 164 | Israel | 31.87(36.86, 33.49) | Croatia | 31.59(54.13, 28.82) | Indonesia | 32.63(38.18, 34.65) |
| 165 | South Africa | 31.75(42.06, 25.44) | Honduras | 31.26(37.54, 34.03) | South Africa | 32.32(43.72, 24.9) |
| 166 | Armenia | 31.57(32.15, 25.95) | South Africa | 30.95(43.71, 25.34) | Armenia | 32.2(37.43, 26.88) |
| 167 | Honduras | 31.5(38.27, 33.12) | Eritrea | 30.73(54.63, 36.15) | Ireland | 31.95(36.96, 35.43) |
| 168 | Croatia | 31.4(50.45, 28.44) | Senegal | 30.37(46.51, 44.71) | Croatia | 31.75(55.57, 29.44) |
| 169 | Senegal | 30.99(43.07, 42.45) | Israel | 30.07(37.05, 33.84) | Honduras | 31.74(37.97, 34.26) |
| 170 | Ireland | 30.66(34.63, 34.04) | Bosnia and Herzegovina | 30.06(52.66, 40.33) | Luxembourg | 31.49(42, 26.29) |
| 171 | Luxembourg | 30.6(35.51, 23.66) | Luxembourg | 30.03(37.76, 23.01) | Senegal | 31.32(44.68, 44.28) |
| 172 | Eritrea | 30.36(54.87, 34.33) | Armenia | 29.83(32.87, 24.42) | Bosnia and Herzegovina | 31.12(54.27, 40.52) |
| 173 | Bosnia and Herzegovina | 30.34(50.58, 38.3) | Ireland | 29.68(35.19, 33.08) | Kyrgyzstan | 30.24(31.27, 36.68) |
| 174 | Kyrgyzstan | 29.98(28.14, 35.27) | Tajikistan | 29.1(37.17, 42.11) | Tajikistan | 30.14(40.07, 43.76) |
| 175 | Tajikistan | 29.87(35.8, 41.62) | Kuwait | 28.9(35.85, 28.36) | Eritrea | 29.97(54.27, 35.62) |
| 176 | Kuwait | 29.07(36.61, 28.37) | Kyrgyzstan | 28.77(27.47, 34.66) | Comoros | 29.05(44.94, 33.66) |
| 177 | Comoros | 28.86(46.44, 31.91) | Comoros | 28.33(47.68, 34.27) | Kuwait | 29.05(36.24, 29.4) |
| 178 | Egypt | 27.83(33.17, 34.83) | Liberia | 27.26(50.4, 22.5) | Sweden | 28.96(28.51, 29.18) |
| 179 | Sweden | 27.42(26.95, 28.55) | Egypt | 27.13(37.47, 34.62) | Egypt | 28.67(38.07, 36.54) |
| 180 | Georgia | 26.99(42.42, 21.27) | Georgia | 26.27(45.61, 21.85) | Georgia | 27.59(51.41, 23.22) |
| 181 | Lithuania | 26.18(34.56, 24.09) | Sweden | 25.46(27.03, 26.6) | Lithuania | 27.22(37.04, 24.92) |
| 182 | Liberia | 24.41(50.89, 21.23) | Lithuania | 24.75(34.96, 22.98) | Norway | 26.36(56.82, 19.51) |
| 183 | Norway | 24.04(48.28, 18.01) | Norway | 23.48(53.61, 16.14) | Spain | 24.88(55.28, 18.02) |
| 184 | Spain | 23.35(50.52, 16.81) | Spain | 22.49(53.28, 15.9) | Liberia | 24.68(49.79, 21.6) |
| 185 | Iceland | 20.92(39.48, 21.28) | Iceland | 19.61(45.55, 19.64) | Iceland | 22.98(48.09, 22.91) |

**Supplementary Table 8. Characteristics of Participants in the National Health and Nutrition Examination Survey**

| **Characteristic** | **N = 45,460** |
| --- | --- |
| Number, weighted | 192,344,015 |
| Year, n (unweighted) (%) |  |
| 1999-2000 | 3,569 (7.76%) |
| 2001-2002 | 4,501 (10.01%) |
| 2003-2004 | 4,212 (9.54%) |
| 2005-2006 | 4,252 (9.91%) |
| 2007-2008 | 5,090 (9.98%) |
| 2009-2010 | 5,250 (9.94%) |
| 2011-2012 | 4,449 (10.17%) |
| 2013-2014 | 4,805 (10.61%) |
| 2015-2016 | 4,747 (10.86%) |
| 2017-2018 | 4,585 (11.23%) |
| Age, years, Mean (SE) | 47.21 (0.19) |
| Sex, n (unweighted) (%) |  |
| Female | 23,248 (51.40%) |
| Male | 22,212 (48.60%) |
| Race, n (unweighted) (%) |  |
| Non-Hispanic White | 20,734 (69.54%) |
| Others | 24,726 (30.46%) |
| Marry, n (unweighted) (%) |  |
| Married | 24,049 (56.35%) |
| other | 21,411 (43.65%) |
| Education level, n (unweighted) (%) |  |
| Less than high school | 11,919 (16.69%) |
| High school or equivalent | 10,608 (24.20%) |
| Above high school | 22,933 (59.11%) |
| Smoking status, n (unweighted) (%) |  |
| Never | 24,477 (53.50%) |
| Other | 20,983 (46.50%) |
| Drinking status, n (unweighted) (%) |  |
| Never | 6,465 (11.25%) |
| Other | 38,995 (88.75%) |
| physical activity, MET.min/week, Mean (SE) | 2,679.62 (49.89) |
| PHDI total score, Mean (SE) | 31.96 (0.17) |
| Whole grains score, Mean (SE) | 1.69 (0.02) |
| Whole fruit score, Mean (SE) | 4.43 (0.05) |
| Nonstarchy veg score, Mean (SE) | 4.70 (0.04) |
| Nuts seeds score, Mean (SE) | 1.64 (0.03) |
| Legumes score, Mean (SE) | 0.92 (0.02) |
| Unsaturated fat score, Mean (SE) | 4.69 (0.02) |
| Fish score, Mean (SE) | 1.20 (0.02) |
| Starchy veg score, Mean (SE) | 2.65 (0.02) |
| Dairy score, Mean (SE) | 4.69 (0.02) |
| Red meat score, Mean (SE) | 0.71 (0.01) |
| Poultry score, Mean (SE) | 1.54 (0.02) |
| Egg score, Mean (SE) | 1.67 (0.02) |
| Saturated fat score, Mean (SE) | 0.33 (0.01) |
| Add sugars score, Mean (SE) | 1.11 (0.02) |

All means and standard errors (SEs) for continuous variables and percentages for categorical variables were weighted.
